# Temozolomide-induced guanine mutations create exploitable vulnerabilities of guanine-rich DNA and RNA regions in drug-resistant gliomas

**DOI:** 10.1126/sciadv.abn3471

**Published:** 2022-06-22

**Authors:** Deanna M. Tiek, Beril Erdogdu, Roham Razaghi, Lu Jin, Norah Sadowski, Carla Alamillo-Ferrer, J. Robert Hogg, Bassem R. Haddad, David H. Drewry, Carrow I. Wells, Julie E. Pickett, Xiao Song, Anshika Goenka, Bo Hu, Samuel A. Goldlust, William J. Zuercher, Mihaela Pertea, Winston Timp, Shi-Yuan Cheng, Rebecca B. Riggins

**Affiliations:** 1 The Ken and Ruth Davee Department of Neurology, Lou and Jean Malnati Brain Tumor Institute, and Robert H. Lurie Comprehensive Cancer Center, Northwestern University Feinberg School of Medicine, Chicago, IL 60611, USA.; 2 Department of Oncology, Lombardi Comprehensive Cancer Center, Georgetown University Medical Center, Washington, DC 20057, USA.; 3 Department of Biomedical Engineering, Johns Hopkins University, Baltimore, MD 21218, USA.; 4 Structural Genomics Consortium, UNC Eshelman School of Pharmacy, University of North Carolina at Chapel Hill, Chapel Hill, NC 27599, USA.; 5 Biochemistry and Biophysics Center, National Heart, Lung, and Blood Institute, National Institutes of Health, Bethesda, MD 20892, USA.; 6 UNC Lineberger Comprehensive Cancer Center, UNC Eshelman School of Pharmacy, University of North Carolina at Chapel Hill, Chapel Hill, NC 27599, USA.; 7 John Theurer Cancer Center, Hackensack University Medical Center, Hackensack, NJ 07601, USA.

## Abstract

Temozolomide (TMZ) is a chemotherapeutic agent that has been the first-line standard of care for the aggressive brain cancer glioblastoma (GBM) since 2005. Although initially beneficial, TMZ resistance is universal and second-line interventions are an unmet clinical need. Here, we took advantage of the known mechanism of action of TMZ to target guanines (G) and investigated G-rich G-quadruplex (G4) and splice site changes that occur upon TMZ resistance. We report that TMZ-resistant GBM has guanine mutations that disrupt the G-rich DNA G4s and splice sites that lead to deregulated alternative splicing. These alterations create vulnerabilities, which are selectively targeted by either the G4-stabilizing drug TMPyP4 or a novel splicing kinase inhibitor of cdc2-like kinase. Last, we show that the G4 and RNA binding protein EWSR1 aggregates in the cytoplasm in TMZ-resistant GBM cells and patient samples. Together, our findings provide insight into targetable vulnerabilities of TMZ-resistant GBM and present cytoplasmic EWSR1 as a putative biomarker.

## Teaser

Genome-wide analysis identifies non-coding, drug-induced nucleotide changes as vulnerabilities in drug resistant glioblastoma.

## INTRODUCTION

Glioblastoma (GBM) is the most diagnosed glioma and has an abysmal overall survival (OS) rate of ~5% at 5 years (*1*). With a median survival time of 14 to 16 months, it is uniformly fatal (*2*). Temozolomide (TMZ; Temodar) is the U.S. Food and Drug Administration (FDA)–approved standard of care first-line therapy for GBM in combination with surgery and radiation (*3*). TMZ causes DNA damage by adding mutagenic adducts to DNA, predominantly *O*^6^-methylguanine, a lesion that can be repaired by the suicide DNA repair protein methyl guanine methyl transferase (MGMT) (*4*). The OS benefit provided by TMZ is ~4 months, and rapid development of TMZ resistance occurs at least in part through demethylation of the *MGMT* promoter, allowing for the expression of MGMT (*5*).

TMZ preferentially targets guanines (*6*), critical nucleotides in many DNA and RNA secondary structures (*7*). Two structurally and functionally important and distinct G-rich regions are G-quadruplexes (G4s) and RNA splice sites. Previous studies have shown G4s to be important regulatory elements for oncogenes like *c-MYC*, *KIT*, or *KRAS* (*7*). These G4s have diverse structural and physicochemical properties that suggest a high degree of selectivity, and thus, G4s may represent a selective druggable target in multiple cancers (*8*). In contrast, studies of *C9orf72* in amyotrophic lateral sclerosis (ALS), a neurodegenerative disease (ND), show that alterations of G4 tracts can cause nucleolar stress, deregulate RNA alternative splicing (AS), and alter RNA binding protein localization (*9*). Nucleolar stress, manifested as changes in nucleolar size and circularity, is a poor prognostic factor in many cancers, including GBM (*10*).

Changes in RNA AS patterns are widespread in GBM, with prior work suggesting that GBM is “addicted to splicing” (*11*). Previous work has shown that targeting splicing signatures may be effective in certain types of gliomas. Braun *et al.* (*11*) showed that gliomas have a high propensity to accumulate detained introns, which can be targeted through Protein Arginine Methyltransferase 5 (PRMT5) inhibition. Other splicing families, like the serine/arginine rich (SR) family and cdc2-like kinases (CLKs), have also been shown to play modulatory roles in specific oncogenic processes (*12*). One family with essential functions in RNA processing in cancer and NDs is the FET family of proteins, consisting of FUS, EWSR1, and TATA-Box Binding Protein Associated Factor 15 (TAF15) (*13*). The aggregation-prone properties of the FET proteins have established FUS as a prototype to study protein aggregation, and a biomarker for ALS and frontotemporal dementia where EWSR1 has similar aggregation-prone properties (*14*). FET proteins also are involved in DNA repair (*15*), can bind the G4 secondary structure of DNA and RNA, and play roles in AS isoform fate (*16*).

In this study, we sought to use the well-known mechanism of action of TMZ to define targetable dependencies of TMZ-resistant GBM. We went on to exploit the TMZ-induced DNA and RNA changes in TMZ-resistant GBM with both known and novel small molecules targeting G4s and the activity of splicing regulatory kinases. Last, we propose EWSR1 cytoplasmic aggregation as a putative biomarker for TMZ-resistant GBM where AS modulators may be successful second-line interventions.

## RESULTS

### TMZ-induced guanine mutations

**Fig. 1.**
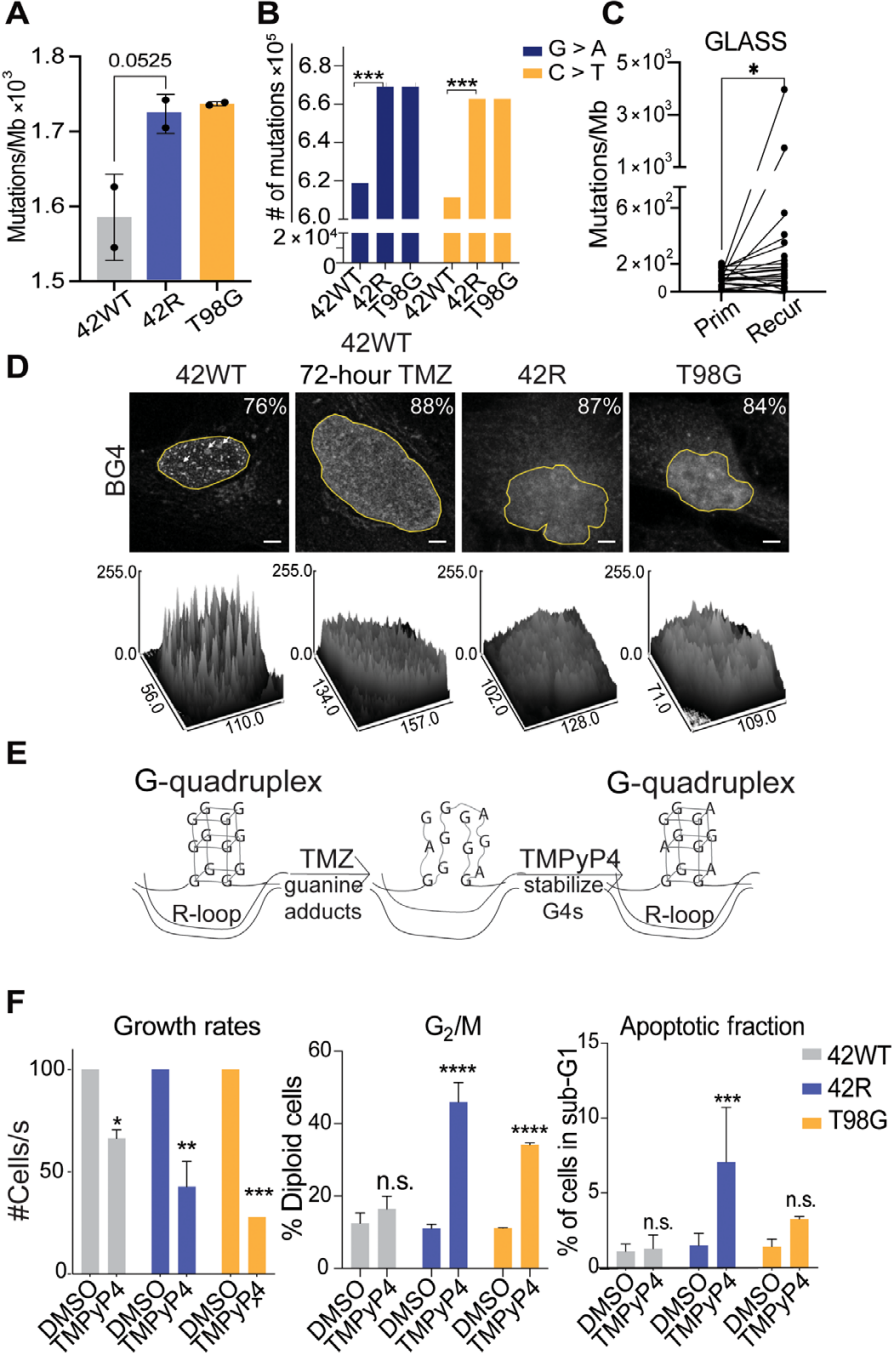
Guanine mutations in TMZ-resistant GBM cells disrupt G4 structure and associate with sensitivity to the G4-stabilizing drug TMPyP4. (**A**) Mutations/Mb identified by WGS data aligned to GRCh38 and analyzed by Genome Analysis Toolkit (GATK) mutation calling; duplicate samples with two-way analysis of variance (ANOVA) for total mutation count. (**B**) Enrichment of G > A and C > T transition mutations in TMZ-resistant cells from data in (A); duplicate samples with two-way ANOVA as in (A). (**C**) Mutations/Mb identified from the GLASS cohort of matched primary (Prim) and recurrent (Recur) samples. (**D**) Immunofluorescence (IF) staining with the G4 antibody BG4 in TMZ-sensitive 42WT, 72-hour–treated 42WT, TMZ-resistant 42R, and T98G cells with surface plots of signal intensity below generated by FIJI. Scale bars, 5 μm. Yellow outline denotes the nucleus and the region analyzed in FIJI; representative of three biological replicates, *n* = 35 cells per replicate per cell line with calculated percentages in the corner. (**E**) Graphical depiction of the hypothesis that TMZ-induced *O*^6^-methylguanine adducts and subsequent mutations disrupt G4s and that the G4 stabilizing agent TMPyP4 may normalize G4 structure. (**F**) Fluorescence-activated cell sorting (FACS) analysis following 24 hours of treatment with 50 μM TMPyP4 or dimethyl sulfoxide (DMSO) vehicle control in 42WT, 42R, and T98G cells; three biological replicates. Growth rates, *t* test; percent of cells in G_2_/M, *t* test; percent of cells with sub-G1 DNA content, *t* test. *P < 0.05, **P < 0.001, ***P < 0.0005, and ****P < 0.0001. n.s., not significant.

TMZ (Temodar), the FDA-approved standard of care first-line therapy for GBM, adds mutagenic adducts to guanine, the most prevalent being *O*^6^-methylguanine. To determine the role of guanine (G) mutations in TMZ resistance, we performed whole-genome sequencing (WGS) on patient-derived GBM cell lines that are TMZ sensitive (42WT), which have acquired resistance to TMZ through an in vitro selection (42R) (*17*), or the established, intrinsically TMZ-resistant GBM line T98G. Acquired TMZ-resistant GBM cells had significantly increased mutational burden relative to their isogenic TMZ-sensitive cells (Fig. 1A), while intrinsic TMZ-resistant GBM cells have a mutational burden comparable to the acquired TMZ-resistant cells. Further analysis of these mutational differences showed that G > A and C > T point mutations were the most enriched in TMZ-resistant cells (Fig. 1B). These results are consistent with the previously defined signature 11 (increased C > T point mutations; fig. S1 for full signature profiles) that is enriched in GBM patient samples and has a probable association with TMZ treatment (*18*). Furthermore, analysis of the Glioma Longitudinal Analysis (GLASS) Consortium data also showed an increase in tumor mutation burden between matched primary and resistant patient samples (Fig. 1C), with an enrichment of C > T mutations (fig. S1).

Two G-rich regions that are functionally and structurally important in cancer are G4s and RNA splice sites. G4s can be readily detected by bioinformatic analysis of WGS data. To test the hypothesis that G4 structures would be perturbed in TMZ-resistant cells, we used Quadron, an algorithm that integrates sequence information to detect tracts of Gs and Cs with characteristic surrounding sequence features of G4s (*19*). After all G4s were detected, we identified G4 sequences unique to each cell line, and these unique G4s were then used to identify significantly enriched motifs using Multiple Expectation maximizations for Motif Elicitation (MEME) (*20*). In TMZ-sensitive 42WT cells, the top three significantly enriched G-rich sequences contain solely Gs as hypothesized (fig. S2A). However, in the acquired resistant 42R cells, we found enrichment of a sequence with higher representation of As and Ts (fig. S2A). The T98G line, which is intrinsically resistant and was not selected in culture with TMZ treatment, showed enrichment of C- and G/A-rich motifs (fig. S2A). These potential structural changes caused by mutations in G4s in the TMZ-resistant cells were then validated by immunofluorescence (IF) with the G4-detecting monoclonal antibody BG4. In the TMZ-sensitive 42WT cells, discrete nuclear puncta were detected by the BG4 antibody, in addition to more diffuse nucleolar staining (Fig. 1D). Confirming prior studies (*21*) showing that BG4 specifically binds DNA G4s in vivo, treatment of 42WT cells with deoxyribonuclease I (DNase I) abrogated specific nuclear BG4 staining (fig. S2B). In the acute TMZ-treated 42WT cells and both TMZ-resistant cell lines, there were no longer discrete G4 nuclear puncta, which corroborated our WGS data (Fig. 1D). This was further confirmed in a second TMZ-sensitive/resistant isogenic pair (8WT/R) showing a decrease of punctate BG4 staining in the TMZ-resistant cell line (8R) (fig. S2E). We then hypothesized that if disruption of G4 sequences played a functional role in TMZ resistance, then a G4-stabilizing drug may be more effective as a second-line treatment in TMZ-resistant cells (Fig. 1E). Treatment with TMPyP4, a G4-stabilizing drug, caused a slight increase in the punctate BG4 staining pattern in 42R cells (fig. S2C). In both resistant cell lines, TMPyP4 treatment induced a significant G_2_/M arrest and decreased cell proliferation with increased sub-G1 fraction only in the 42R resistant cell line but had minimal effects on cell cycle profile and a slight decrease in growth in the TMZ-sensitive cell line (Fig. 1F). Further extending these data, we tested TMPyP4 in normal human astrocytes (NHAs), a second TMZ-sensitive and TMZ-resistant paired line (8WT and 8R), as well as independent TMZ-sensitive (U87) and TMZ-resistant (U118) GBM lines (*22*) where only the TMZ-R lines showed a decrease in growth (fig. S2F). Furthermore, *ATRX* mutations have been tabulated between the TMZ-sensitive and TMZ-resistant models as *ATRX* deficiency can also affect G4-targeted therapy (table S3) (*23*). These data support the hypothesis that G4-disrupting G mutations have a functional, and potentially targetable, role selectively in TMZ-resistant GBM.

### Acquired nucleolar changes with TMZ-induced mutations in G4s

**Fig. 2.**
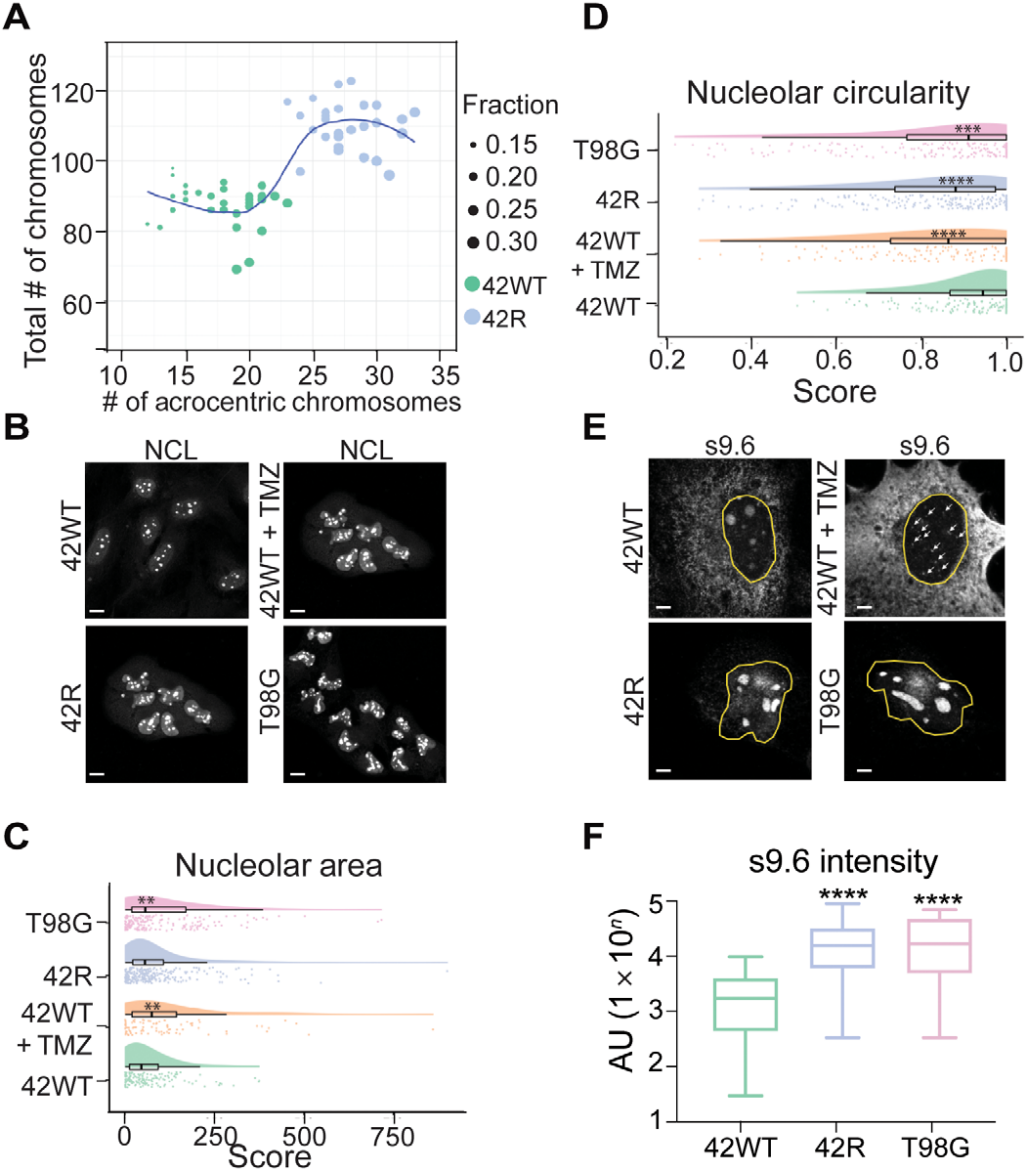
TMZ treatment and resistance associate with increased nucleolar stress and the formation of nucleolar R-loops. (**A**) Analysis of acrocentric chromosome fraction between 42WT and 42R in metaphase spreads (42WT, *n* = 31; 42R, *n* = 27) from each cell line; *t* test ****. (**B**) Representative images of endogenous nucleolin (NCL) IF. (**C**) Quantification of nucleolar area from images in (B). (**D**) Quantification of nucleolar circularity from images in (B). IF with the R-loop binding S9.6 antibody in (**E**) 42WT, 72-hour TMZ treatment in 42WT, 42R, and T98G. (**F**) Quantification of R-loop intensity in (E). Scale bars, 50 μm (B) and 5 μm (E). AU, arbitrary units. **P < 0.001, ***P <0 .0005, and ****P < 0.0001.

G4s are abundant in nucleoli, major stress organelles in cells undergoing DNA damage, where nucleoli morphology is a poor prognostic marker in several cancers, including GBM (*10*). Nucleoli form around nuclear organizer regions located on the p arm of the five acrocentric chromosomes: 13, 14, 15, 21, and 22. Analysis of metaphase spreads from 42WT versus 42R cell lines showed an increase in total chromosome number in the 42R cell line (fig. S3A) (*17*). We reanalyzed these data to determine whether the acrocentric, or nucleolar-forming, chromosomes were specifically hyperenriched in the acquired resistant cell line and showed that the 42R cells had a significantly higher fraction of acrocentric chromosomes compared to their TMZ-sensitive parental line (Fig. 2A). Furthermore, we identified a few patients from the GLASS consortium whose recurrent tumors showed an increased log_2_ copy ratio (>/= 2) in acrocentric chromosomes 13, 14, and 21 (fig. S3B). Next, we adapted the index of nucleolar disruption score as a method of quantifying nucleolar stress that accounts for both size and roundness of nucleoli (*10*). Using IF staining and automated analysis of the nucleolar protein nucleolin (NCL) to determine nucleolar size and circularity (Fig. 2B), we found significant increases in nucleolar size associated with both acute TMZ treatment (42WT + TMZ) and intrinsic resistance (T98G; Fig. 2C) and significant decreases in circularity or nucleolar roundness in acute TMZ-treated and both acquired and intrinsic TMZ-resistant nucleoli (Fig. 2D).

Within nucleoli, RNA polymerase I transcribes ribosomal RNAs (rRNAs), which can hybridize to antisense ribosomal DNA (rDNA) to form rRNA:rDNA R-loops (*24*). We used the R-loop–specific antibody S9.6 to visualize nucleolar R-loop changes (*25*). The TMZ-sensitive 42WT cells showed discrete, DNA damage–induced punctate R-loop accumulation throughout the nucleus (*26*) when treated with TMZ (Fig. 2E), compared to the nucleolar staining in dimethyl sulfoxide (DMSO)–untreated 42WT cells (Fig. 2E). However, TMZ-resistant cell lines showed a robust increase in nucleolar R-loop accumulation rather than discrete nucleoplasmic puncta associated with a normal DNA damage response, as reported by Gorthi *et al*. (*26*) (Fig. 2, E and F). We validated the specificity of the R-loop antibody, S9.6, by treating cells with ribonuclease H (RNase H), which digests R-loops. This treatment abrogated specific nucleolar staining (fig. S2D). Overall, TMZ-resistant GBM cells exhibit a nucleolar stress response with an increase in the number of acrocentric chromosomes, nucleolar size and circularity changes, and differential R-loop staining patterns.

### Isoform usage changes in TMZ-resistant cells

**Fig. 3.**
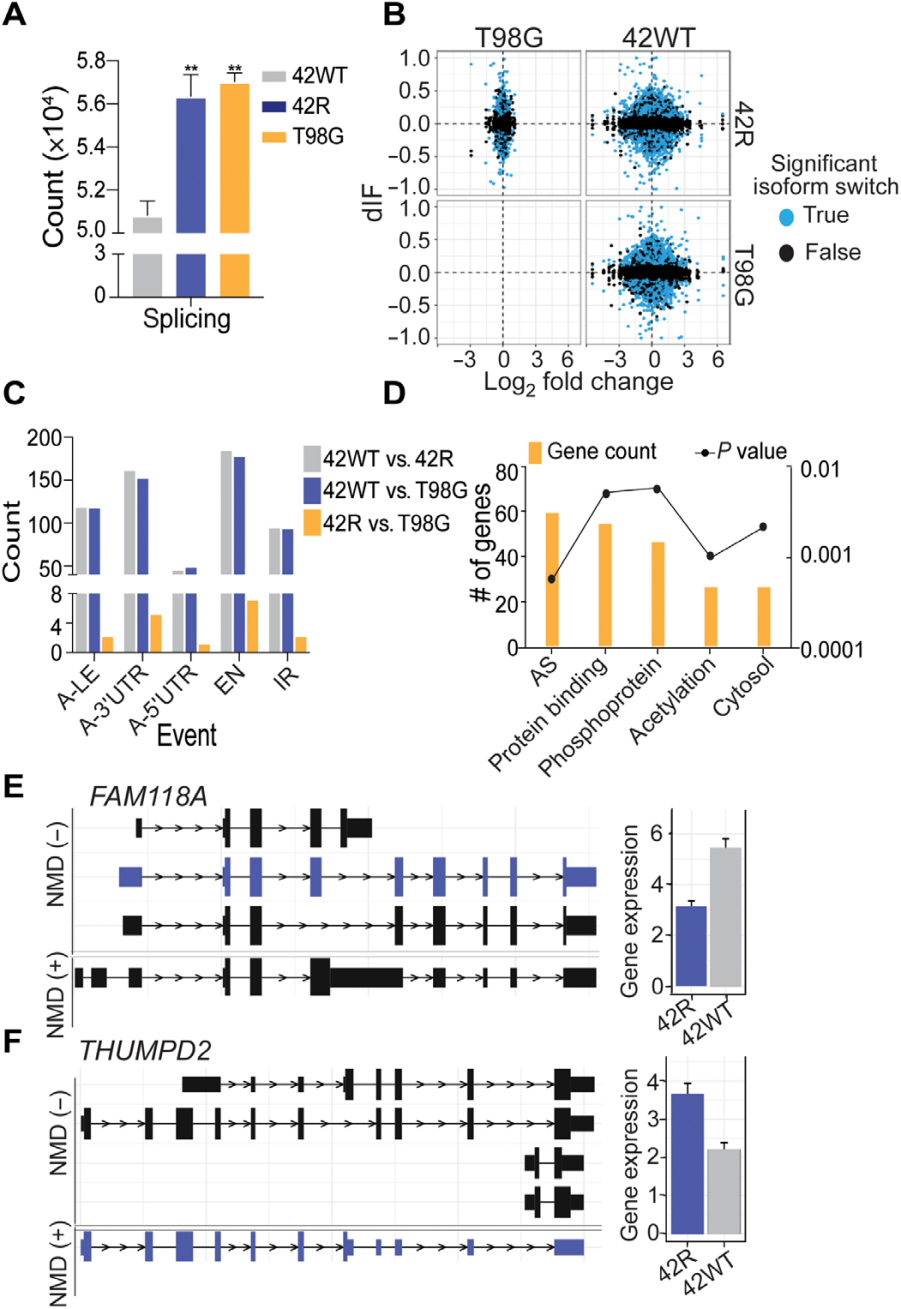
Splice-site mutations in TMZ-resistant GBM cells associate with increased exon and isoform usage changes in TMZ-resistant cells. (**A**) Quantification of splice-site mutations in TMZ-sensitive versus TMZ-resistant cells from WGS analyses described in Fig. 1; one-way ANOVA. **P < 0.001. (**B**) Nanopore full-length cDNA sequencing analysis with Illumina short-read correction to determine the gene expression changes (*x* axis) as compared to isoform usage changes (*y* axis; dIF, differential isoform fraction). (**C**) Nanopore analysis of AS type, where the events are not mutually exclusive. A-3′UTR, alternative 3′ untranslated region; A-5′UTR, alternative 5′ untranslated region; A-LE, alternative last exon; EN, exon number change; IR, intron retention. (**D**) DAVID pathway analysis of gene subset which had both a gene isoform usage switch and annotated splicing mutation. (**E**) *FAM118A* and (**F**) *THUMPD2* gene isoform changes between nonsense-mediated decay (NMD)–sensitive and NMD-insensitive in TMZ-sensitive (42WT) versus TMZ-resistant (42R) cells.

WGS analysis further identified a significant increase in mutations in G-rich splice sites in TMZ-resistant cells (Fig. 3A). Splicing mutations and altered splicing patterns are characteristic of many cancers, including GBM (*27*). To quantify changes in categories of splicing events in the TMZ-resistant versus TMZ-sensitive cells, we performed nanopore cDNA sequencing to interrogate full-length transcripts, followed by Illumina short-read validation. The Albacore workflow was used to call cDNA bases, followed by long-read alignment with minimap2 (*28*). Then, both IsoformSwitchAnalyzeR (*29*) and SUPPA2 (*30*) pipelines were used to identify changes in the differential gene isoform usage, between TMZ-sensitive and TMZ-resistant cells, even in cases where there was no difference in overall gene expression (Fig. 3B). As other studies have investigated differentially expressed genes, we prioritized interrogation of genes with differential isoform usage. We first compared the switched gene expression between all three cell lines and found the highest overlap in switched gene expression between T98G versus 42WT and 42R versus 42WT, suggesting more common changes with resistance acquisition (fig. S4A). We then analyzed the type of splicing changes and found differential exon number (EN) to be the top IsoformSwitchAnalyzeR-annotated change (Fig. 3C). Next, we tested the occurrence of mutations within the isoform-switched genes (121 of 153) as compared to nonswitching genes (17,815 of 32,542), where Fisher’s exact test showed a significant enrichment of mutations correlating to switched genes (*P* = 3.12 × 10^−10^). Looking at the point mutation types in the switched genes, we again found the highest number of G➔A and C➔T mutations in these switched genes (fig. S4B). A group of 94 isoform-switched genes that contained G mutations were selected for further downstream analysis (table S1). These 94 were annotated and interrogated in the GLASS dataset where 26 genes also had these G > A or C > T splice site mutations in matched recurrent versus primary tumor patient samples (fig. S4, C to E). We then assessed global pathway changes via DAVID (*31*) to determine potential druggable vulnerabilities in the 94 isoform-switched genes with G mutations. We found an enrichment of genes associated with AS, protein binding, phosphorylation, acetylation, and the cytosol (Fig. 3D and table S1). We then interrogated functional changes of the switched isoforms and found a subset of genes that switched from nonsense-mediated decay (NMD) sensitive to insensitive or vice versa. For example, FAM118A switched from a dominant NMD-sensitive isoform [NMD (+)] to an insensitive isoform [NMD (−); purple isoform; WT versus R ∆PSI = 0.33, *P* = 0.04], although overall gene expression shows decreased expression in resistant cells [Fig. 3E and fig. S4F (yellow isoform = NMD − isoform)]. In this way, total gene expression would seem to decrease; however, an NMD-insensitive isoform may have a longer half-life. To this end, *FAM118A* has previously been identified as a potential gene target in glioma stem cells, in which a decrease in FAM118A expression, but increase in FAM118A protein levels, correlates with worse OS of patients with GBM (*32*). On the other hand, THUMPD2 shows an increase in total gene expression (higher purple bar), but a switch to an NMD-sensitive isoform in the TMZ-resistant cells [Fig. 3F, NMD (+); purple isoform; WT versus R ∆PSI = 0.29, *P* = 0.03; fig. S4G; yellow bar total expression, yellow isoform = NMD + isoform]. Here, total gene expression would predict higher expression levels, although this transcript is an NMD target that could lead to a shorter half-life. Consistent with this, decreased THUMPD2 protein expression, but not mRNA, is correlated with chemotherapy resistance (*33*). We further interrogated differential isoform usage between TMZ-sensitive and TMZ-resistant cell lines, as well as GLASS-matched patient samples, and found differential gene isoform usage that correlates with G mutations within these genes.

### CLK2 inhibition preferentially targets TMZ-resistant cells

**Fig. 4.**
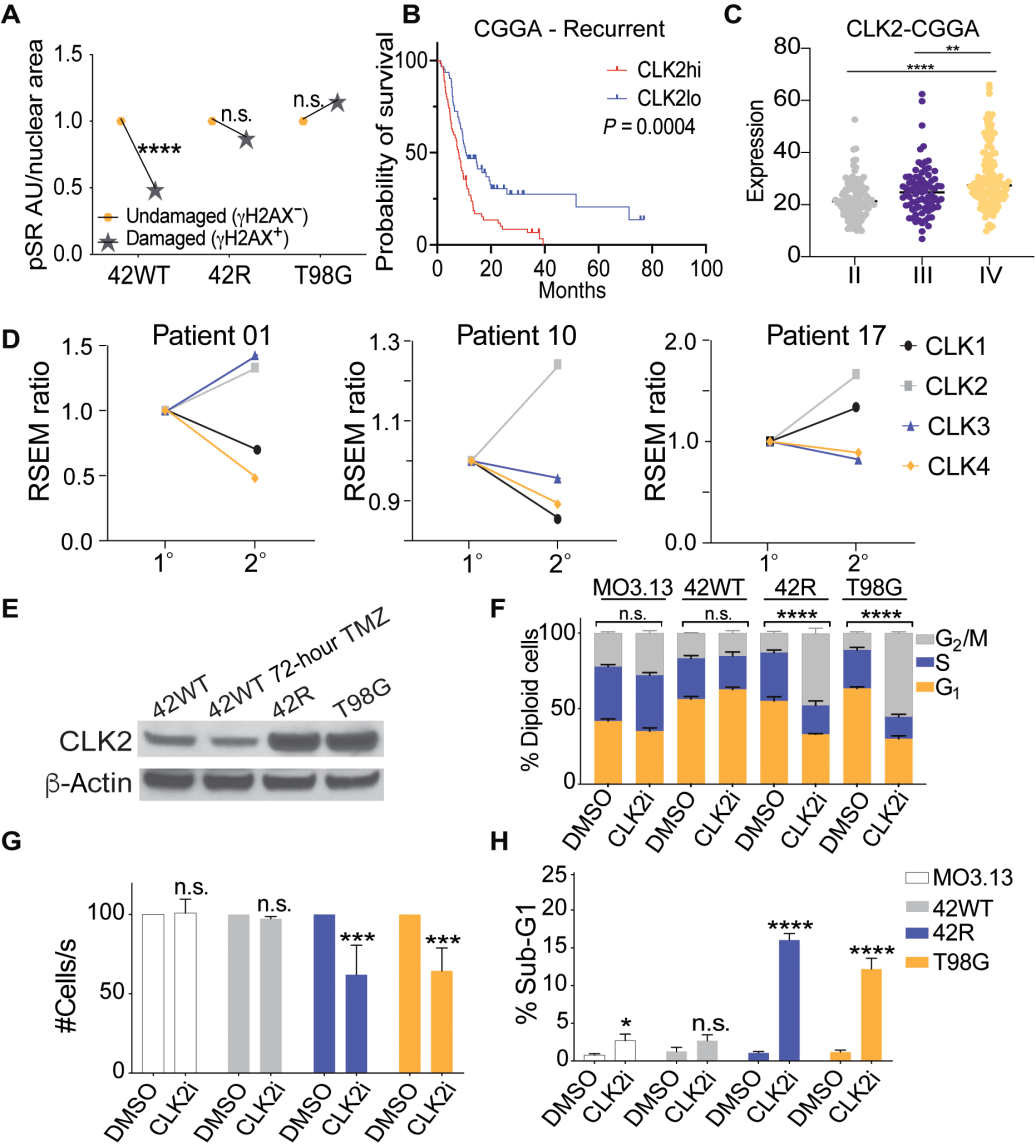
CLK2 inhibition with a novel CLK inhibitor (GW807982X and CLK2i) specifically targets TMZ-resistant GBM cells. (**A**) Quantified IF of pSR (1H4) intensity in TMZ-treated cells with (γH2AX^+^) or without (γH2AX^−^) DNA damage; two-tailed *t* test. (**B**) Association between CLK2 mRNA expression and OS from GlioVis CGGA GBM recurrent patient samples; log-rank (Mantel-Cox) test. (**C**) Association between CLK2 expression and increasing tumor grade from the CGGA dataset; one-way ANOVA. (**D**) Change in RNA sequencing RPKM of *CLK-1* to *CLK1-4* in three matched patient samples from primary (1°) GBM and post-TMZ treatment (2°) (*71*). *P < 0.05, **P < 0.001, ***P < 0.0005, and ****P < 0.0001. (**E**) Western blot of CLK2 expression in GBM TMZ-sensitive, TMZ-treated, and TMZ-resistant cell lines. (**F**) FACS analysis for cell cycle profile in response to 5 μM CLK2i versus DMSO vehicle control treatment for 24 hours; three biological replicates with one-way ANOVA. (**G**) Relative cell numbers following 24-hour treatment with 5 μM CLK2i versus DMSO vehicle control; three biological replicates with *t* test. (**H**) Sub-G1 or apoptotic DNA content in cells analyzed in (G); three biological replicates with *t* test. RSEM, RNA-Seq by Expectation-Maximization; RPKM, Reads Per Kilobase of transcript, per Million mapped reads.

As our gene isoform usage analysis found substantial changes in EN and AS pathway regulation, we sought to functionally target RNA splicing in the TMZ-resistant cells. Several splicing factor families drive exon inclusion relative to exclusion events, including SR proteins. SR protein activity and localization are dependent on their phosphorylation status, which can be altered by DNA damage (*34*). We found that TMZ treatment induced a significant decrease in the phosphorylation of SR proteins (pSRs) only in TMZ-sensitive cells exhibiting DNA damage, as measured by the presence of the double-strand break marker γH2AX (Fig. 4A). Dephosphorylation of pSRs was not observed in either TMZ-resistant (42R and T98G) cell line post-TMZ treatment (Fig. 4A) or in a second matched TMZ-sensitive/resistant pair (8WT and 8R; fig. S5A), suggesting altered pSR function in TMZ-resistant cells. pSRs can bind both introns and exons to determine isoform fate through exon inclusion or exclusion events, and SR proteins that are not hyperphosphorylated are less competent to dictate gene isoform fate (*35*). Therefore, we sought to phenocopy the decrease in pSRs caused by TMZ treatment in the sensitive cells by inhibiting a pSR upstream regulator in TMZ-resistant cells. Serine-arginine protein kinase 1 or the CLK family can catalyze the nuclear hyperphosphorylation of multiple SR proteins (*36*). While SRPK1 expression is not associated with outcome in GBM (fig. S5B), analysis of the GlioVis Chinese Glioma Genome Atlas (CGGA) dataset shows a significant association of higher expression of CLK2, but not CLK1, CLK3, or CLK4, with poor OS, and CLK2 mRNA expression significantly increases with grade in multiple brain cancers, including GBM (Fig. 4, B and C). To further confirm increased CLK2 expression post-TMZ treatment, we reanalyzed patient samples from a cohort where three patients had matched RNA sequencing data from pre/post-TMZ–treated tumors. All three recurrent samples had consistent increases of CLK2 expression, while the other CLK family members had varying changes in expression (Fig. 4D). Consistent with this, our acquired and intrinsic TMZ-resistant GBM cells showed a robust increase in CLK2 protein expression as compared to the TMZ-sensitive line (Fig. 4E), but acute TMZ treatment of TMZ-sensitive cells did not increase CLK2 protein expression (Fig. 4E). No CLK2 mutations were observed in TMZ-sensitive versus TMZ-resistant WGS, although significantly increased usage of an NMD-insensitive (NMD−) isoform was observed in the nanopore data (fig. S5, C and D). Together, these data led us to select CLK2 inhibition as a potential strategy to phenocopy in TMZ-resistant cells the decrease in pSRs caused by TMZ in sensitive cells.

GW807982X, here referred to as CLK2i, was published as part of the GSK published kinase inhibitor set (*37*) and identified as a CLK1, CLK2, and CLK4 inhibitor. We further characterized this molecule in orthogonal assay systems and confirm potent and selective CLK inhibition, with an increased specificity for CLK2, using in-cell target engagement assays. The compound is now a part of the kinase chemogenomic set (*38*). Treatment of TMZ-resistant cells with CLK2i led to a decrease in SR phosphorylation at 30 min (fig. S6, A to E) (*39*–*41*) and caused significant G_2_/M arrest, decreased cell proliferation, and increased apoptosis selectively in TMZ-resistant cells (Fig. 4, F to H). CLK2i treatment did not cause significant changes to growth rates or cell cycle and only a modest increase in apoptosis, in the immortalized noncancerous brain oligodendrocyte cell line, MO3.13 (Fig. 4, E to G). Expanding these studies to NHAs, a second TMZ-sensitive/resistant isogenic cell line pair (8WT/8R), independent TMZ-sensitive (U87) and TMZ-resistant (U118) cells, and the glioma stem cell GSC1478 lines, CLK2i treatment consistently showed a greater decrease in cell growth in the TMZ-resistant lines (fig. S6F). These data support a model in which TMZ-resistant GBM cells have rewired their splicing regulatory machinery over time, exhibiting deregulation of SR protein phosphorylation and CLK2 function, and show that pharmacological inhibition of CLK2 may selectively target these alterations, leading to decreased cell proliferation and increased cell death.

### EWSR1 cytoplasmic localization in TMZ-resistant cells and GBM clinical specimens

**Fig. 5.**
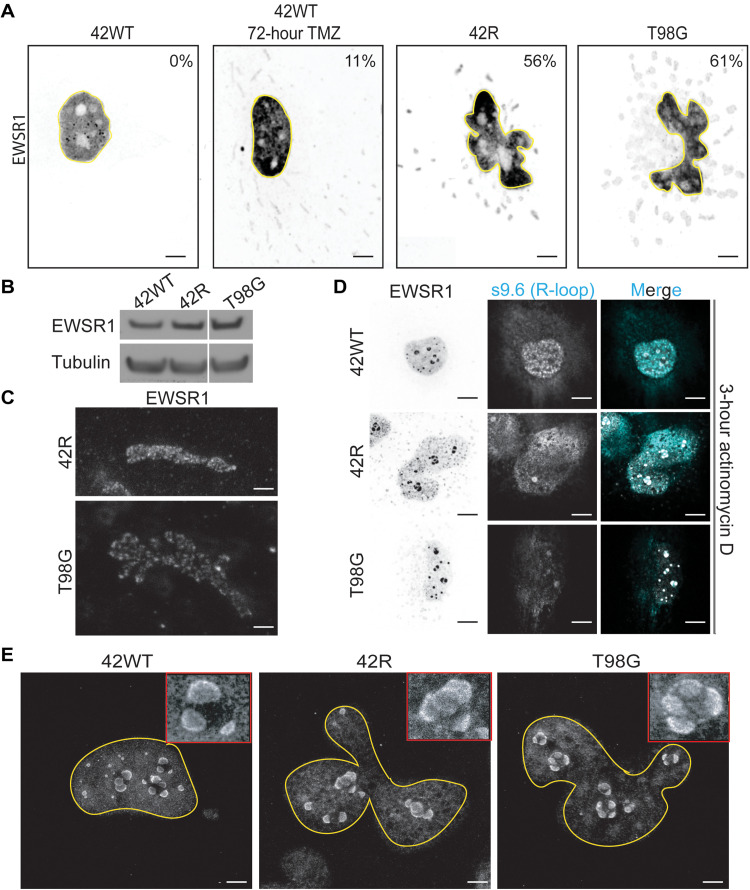
The RNA binding protein EWSR1 forms cytoplasmic, amyloid-like aggregates in TMZ-treated, and TMZ-resistant GBM cells. (**A**) Basal IF of EWSR1 in the indicated cell lines, with the nucleus outlined in yellow. Values indicate percent of cells per condition that show EWSR1 cytoplasmic aggregates. (**B**) Western blot of EWSR1 total protein expression in the indicated cell lines. (**C**) High-resolution STED microscopy imaging of EWSR1 cytoplasmic aggregates in 42R and T98G cells. (**D**) Localization of EWSR1 to nucleoli adjacent to R-loops (S9.6) following 3-hour 10 nM actinomycin D (ActD) treatment to inhibit RNA polymerase I. (**E**) STED imaging of EWSR1 nucleolar movement following 3-hour ActD treatment in 42WT, 42R, and T98G cells. Scale bars, 5 μm (A), 0.5 μm (C), 5 μm (D), and 2.5 μm (E).

Given the collective deregulation of G4s, nucleoli, and splicing observed in TMZ-resistant GBM cells, we sought to identify a robust, independent marker that might be an indicator for TMZ resistance and, potentially, second-line CLK2i efficacy in GBM. EWSR1 is member of the FET family of proteins, which also includes FUS and TAF15, where FUS is an established biomarker in many neurodegenerative disorders (*13*). EWSR1 binds DNA G4 structures (*16*), relocates to nucleoli following ultraviolet-induced DNA damage (*42*), and plays key functional roles in splicing and DNA damage responses (*42*). The nuclear staining of EWSR1 in TMZ-sensitive cells was in marked contrast to both the nuclear and cytoplasmic aggregates seen in acute TMZ-treated and TMZ-resistant cells (Fig. 5A, reversed image to better visualize the cytoplasmic staining). Expression levels of EWSR1 protein did not increase with TMZ resistance (Fig. 5B). The presence of cytoplasmic EWSR1 aggregates in TMZ-resistant cells was validated by a second antibody from a different vendor and host species (fig. S7A). We then used high-resolution stimulated emission depletion (STED) microscopy to better resolve the structure of these aggregates, which showed discrete protein puncta within the cytoplasmic aggregates in both TMZ-resistant cell lines (Fig. 5C), and confirmed that the amyloid-like aggregates were only found in the cytoplasm but not present in the nucleus (fig. S7B). We then performed cell fractionation assays in two pairs of TMZ-sensitive/resistant pairs (42WT/42R and 8WT/8R) and did not see increased abundance of EWSR1 in the cytoplasmic fraction versus nuclear in the TMZ-resistant cell lines (fig. S7C).

Cytoplasmic aggregation of EWSR1 may suggest a potential role for RNA buffering, a defining feature of mislocalized RNA binding proteins in NDs (*43*), where lower RNA concentration in the cytoplasm has been hypothesized to allow the aggregation of these proteins. As EWSR1 has already been shown to relocate to the nucleoli under stressed conditions (*44*), we sought to decrease the local nucleolar RNA concentration and cause a stress response with low-dose actinomycin D (ActD) treatment, testing both an alternative (non-TMZ) stress response and the potential role of RNA in EWSR1 aggregation. Low-dose ActD treatment led EWSR1 to accumulate around the nucleoli, indicated by S9.6 staining for R-loops (Fig. 5D). STED imaging of these samples better resolved EWSR1, with 42WT cells showing a horseshoe-shaped staining pattern in which EWSR1-containing structures did not link to each other (Fig. 5E, left). By contrast, EWSR1 formed a linked structure between the droplets in both the acquired and intrinsic TMZ-resistant cells (Fig. 5E, middle and right). This further suggested a role for RNA buffering in EWSR1 aggregation in TMZ-resistant cells. In addition, dual staining of EWSR1 and DNA G4s (BG4) showed colocalization in the TMZ-sensitive cell line that was not observed in TMZ-resistant cells (fig. S8), further suggesting that the deregulation of G4s identified in TMZ-resistant cells (Fig. 1C) can disrupt the localization of G4-binding proteins like EWSR1. However, EWSR1 aggregates do not appear to drive or maintain TMZ resistance, as knockdown of EWSR1 had minimal effects on cell cycle profile or proliferation in TMZ-resistant cells (fig. S9, A to C). We used leptomycin B, an inhibitor of the nuclear export protein chromosomal maintenance 1, as a control to show that EWSR1 was efficiently trafficked to and from the cytoplasm in both TMZ-sensitive and TMZ-resistant lines (fig. S9D). Ectopically expressed, yellow fluorescent protein–tagged EWSR1 caused nonendogenous ribbon-like formation of EWSR1 in the cytoplasm in the TMZ-resistant cell lines, preventing further overexpression experiments (fig. S9E). Together, these results suggest that EWSR1 is the first, to our knowledge, aggregating RNA binding protein in GBM, where RNA buffering and deregulation of G4 structures may play a role in its aggregation.

**Fig. 6.**
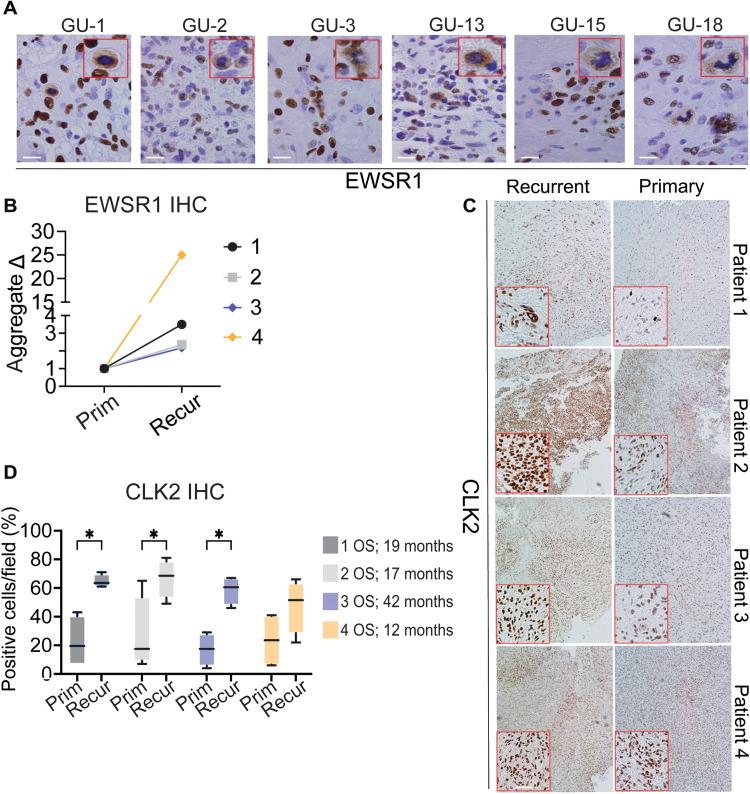
Cytoplasmic EWSR1 aggregates are detected in GBM clinical specimens. (**A**) EWSR1 IHC of six representative GBM patients with cytoplasmic aggregation of EWSR1 (red box inset). (**B**) Quantification of EWSR1 aggregative positive cells between primary (Prim) and recurrent (Recur) matched patient samples. (**C**) Representative images of CLK2 IHC from matched patient samples. (**D**) Quantification of CLK2-positive cells between primary and recurrent matched patient samples from images in (C). OS in months. Scale bars, 20 μm (A) and 100 μm (D). *P < 0.05.

A key limitation of studies conducted in cell culture is their dependence on ex vivo models that have been in culture for decades. To determine whether EWSR1 cytoplasmic aggregates were also present in GBM clinical specimens, we stained a cohort of 21 brain tumor samples, including 15 GBMs, for EWSR1. Nine of the 15 GBM samples were positive for EWSR1 cytoplasmic amyloid–like aggregates (Fig. 6A and table S2, cohort 1), while none of the anaplastic astrocytomas or medulloblastomas showed this staining pattern. To further test our hypothesis that TMZ-resistant disease has an increase of EWSR1 cytoplasmic aggregates and CLK2 expression, we performed immunohistochemistry (IHC) on an independent set of four matched patient samples from pre-TMZ treatment to post–TMZ-resistant recurrent GBM (table S2, cohort 2). We found an increase in EWSR1 cytoplasmic staining in all four samples, with the greatest relative increase observed in the patient with the shortest OS (Fig. 6B). We also observed a significant increase in CLK2 expression in three of the four samples of post–TMZ-resistant recurrent disease, with the fourth sample showing a high baseline CLK2 expression and the lowest OS (Fig. 6, C and D). Overall, these data establish a clinically relevant framework for future studies to determine the functional role of EWSR1 aggregation and aberrant splicing via CLK2 protein abundance in GBM.

## DISCUSSION

Since the pivotal 2005 clinical trial that changed GBM standard of care to the Stupp regimen—adjuvant TMZ post-surgery and radiation—TMZ has been almost exclusively used in brain cancers, despite adding only ~4 months to median OS (*45*). Given the lack of successful FDA-approved second-line treatments for GBM, it is essential to identify pathways that could be targeted in TMZ-resistant GBM (*46*). However, TMZ is rarely used in other cancers, and the 400+ clinical trials to date that have attempted to repurpose chemotherapeutic or targeted agents from other cancers have failed to demonstrate the benefit of these approaches in GBM (*47*). We therefore looked to other central nervous system (CNS) disorders for insight. Mechanistic studies in CNS disorders, including NDs, show pathological disruption of DNA and RNA secondary structures (*48*), splicing, and aberrant localization of RNA binding proteins (*49*). We therefore reframed our strategy for addressing TMZ resistance in the context of nucleotide secondary structure, RNA processing, and RNA binding proteins in TMZ-resistant GBM.

As the first anticancer drugs were DNA targeting and still play a major role in cancer treatments, we focused on the downstream effects of TMZ-induced DNA damage at guanine nucleotides (*8*). Guanine is the most readily oxidized base, with the loss of an electron creating a hole that is preferentially targeted by spontaneous DNA damage, an event whose impact is compounded in tracts of guanines (*50*, *51*). The established mechanism of action of TMZ is the addition of a mutagenic *O*^6^-methyl adduct to guanines (*5*). Guanines are critical nucleotides that stabilize many essential DNA and RNA secondary structures and are the target of many other chemotherapeutic agents (*6*). It also has previously been shown that TMZ-treated GBM has a characteristic mutational signature of C > T (*18*). By performing WGS, we found both C > T and G > A mutations to be enriched in TMZ-resistant GBM cells. We focused on two critical G-rich regions—G4s and splice sites—to assess their functional roles in TMZ resistance. We show sequence- and conformation-based changes in G4s by WGS and IF, respectively, in TMZ-sensitive versus acute TMZ-treated and TMZ-resistant GBM cells. We also demonstrate that the G4-stabilizing ligand TMPyP4 is more growth inhibitory in TMZ-resistant lines compared to TMZ-sensitive cells. Previous studies have demonstrated the efficacy of other G4 ligands in multiple cancer cell lines, with CNS-derived models being the most sensitive to these agents (*52*). Therapeutic targeting of G4s in ALS can also decrease toxic hexanucleotide repeat foci and dinucleotide repeat proteins, leading to attenuated disease phenotypes in vitro (*53*). This avenue of targeting nucleotide secondary structure changes after chemotherapy warrants further investigation and will require the development of interventions that can cross the blood-brain barrier, as TMPyP4 cannot (*54*).

Our results reveal that TMZ-resistant GBM cells show an increase in a second critical DNA:RNA secondary structure:nucleolar R-loops. Nucleoli are major stress organelles, and aberrations in their size and circularity are positively correlated with increasing cancer grade (*10*). The increase in nucleolar R-loops that we demonstrate in TMZ-resistant cells is intriguing, as a normal DNA damage response would result in punctate R-loops throughout the nucleus (*26*), which we observed in TMZ-sensitive cells in response to TMZ. However, this pattern is lost in TMZ-resistant cells. In other settings, increases in nucleolar R-loops have been shown to induce genome instability (*55*) and correlate with rDNA mutations and an aberrant DNA damage response (*24*). This further suggests that there may be a reprogrammed DNA damage response in TMZ-sensitive versus TMZ-resistant cells.

We further focused on G-rich splice sites and their potential contribution to aberrant regulation of AS, a hallmark of many cancers including GBM (*56*). We showed that splicing mutations are enriched in the TMZ-resistant cell lines, where nanopore full-length cDNA sequencing also identified marked enrichment of changes in exon usage. The CLK family of splicing regulatory kinases induces the nuclear hyperphosphorylation of SR proteins. We show that SR proteins are dephosphorylated upon TMZ treatment in TMZ-sensitive, but not TMZ-resistant, cells; there is an increase in CLK2 expression in TMZ-resistant cells, and a novel CLK2 kinase inhibitor is more growth inhibitory in TMZ-resistant lines. CLK2 has previously been pharmacologically targeted in *MYC*-driven breast cancer (*12*) and genetically in GBM (*57*). Targeting splicing broadly is an active area of research with clinical trials underway in *SF3B1* mutant cancers and neurological diseases (*58*). Our data suggest that these efforts should be further expanded to TMZ-resistant GBM.

Our data identify EWSR1 as the first, to our knowledge, aggregating RNA binding protein in TMZ-resistant GBM, using two independent models of acquired and intrinsic TMZ-resistant cell lines and GBM clinical samples. This aggregation phenotype is similar to DNA damaged–induced FUS aggregation observed in FUS-ALS motor neurons (*59*). RNA buffering may also play a role in the aggregation phenotype, as we show that inhibition of nucleolar rRNA transcription by acute low-dose ActD treatment can induce ribbon-like strands of EWSR1 around the nucleolus, although the local concentration of EWSR1 would presumably be increasing as well. The last factor that we explored is the ability of DNA secondary structures to alter protein localization. The inability of EWSR1 to bind the mutated G4s in the TMZ-resistant lines suggests that this may affect the normal function and proper regulation of EWSR1 in both splicing and DNA repair. Together, our data suggest potential overlapping functions of the cytoplasmic aggregates of EWSR1 with other NDs, and further studies may provide important insight on specific upstream regulators of DNA damage–induced EWSR1 aggregation in CNS disorders and possibly other DNA-damaging chemotherapy-treated cancers. However, significantly more research needs to be conducted to determine what role DNA and RNA secondary structures, RNA processing, and RNA binding protein aggregates plays in GBM maintenance and TMZ resistance.

In conclusion, this study illustrates a previously unknown relationship between TMZ resistance and altered DNA/RNA structures and how these changes can affect RNA binding protein function and localization. It further provides evidence that these changes may serve as therapeutic vulnerabilities that are targetable in TMZ-resistant glioma. Future research should be conducted to discover whether these results are generalizable to other chemotherapy-resistant cancers.

## MATERIALS AND METHODS

### Cell lines and culturing conditions

Immortalized human oligodendrocyte MO3.13 cells were a gift from A. Taraboletti [Lombardi Comprehensive Cancer Center (LCCC)]. Patient-derived TMZ-sensitive 42MGBA (42WT) and 8MGBA (8WT) cells were provided by J. Toretsky (LCCC), and the de novo TMZ-resistant T98G cell line was from American Type Culture Collection (ATCC). The acquired TMZ-resistant 42R and 8R cell line variant was developed by our laboratory and previously described (*17*). NHAs, U118, U87, and GSC lines were provided by the Cheng Lab. All cells tested negative for *Mycoplasma* contamination and were maintained in a humidified incubator with 95% air:5% carbon dioxide. All cell lines were fingerprinted by the LCCC Tissue Culture and Biobanking Shared Resource (TCBSR) to verify their authenticity using the PowerPlex 16 HS System (Promega, USA). The 42R cells are documented to be of the same origin as their parental cell line. All cell lines were grown in Dulbecco’s modified Eagle’s medium (DMEM; high glucose, Thermo Fisher Scientific, #11965092) with 10% fetal bovine serum (FBS).

### Immunofluorescence

Cells were seeded at a density of 25,000 to 30,000 cells onto 18-mm-diameter #1.5 round coverslips (VWR, #101413-518) in 12-well dishes (day 1). They were allowed to attach to the coverslips for a full day (day 2). On the following day (day 3), the media were removed, and cells were washed three times with phosphate-buffered saline (PBS) and then fixed and permeabilized in 3.2% paraformaldehyde (PFA) with 0.2% Triton X-100 in PBS for 5 min at room temperature. Three washes were performed with PBS in the 12-well plate, and then coverslips were inverted onto 120 μl of primary antibody in the antibody block (0.1% gelatin with 10% normal donkey serum in distilled H_2_O) on strips of parafilm and incubated for 2 hours. Coverslips were first incubated with either BG4 (1:150; Sigma-Aldrich, MABE1126), EWSR1 (rabbit monoclonal antibody; 1:600; Abcam, ab133288; mouse monoclonal antibody; 1:200; SCBT, sc-48404), S9.6 (1:150; Sigma-Aldrich, MABE1095), or NCL (0.02 mg; 1:500; Novus, NBP2-44612) for 2 hours. The donkey serum, nonconfluent cells, 2-hour incubation for primary antibodies, and day 3 staining are vital for visualizing consistent EWSR1 cytoplasmic staining. After incubation with primary antibodies, coverslips were washed three times with PBS. Then, coverslips were inverted onto 100 μl of antibody block with secondary antibodies (Alexa Fluor 488 anti-mouse; 1:200; Life Technologies, #A11029; Alexa Fluor 594 anti-rabbit; 1:200; Life Technologies, #A11037) and 4′,6-diamidino-2-phenylindole (DAPI) (DNA, 1:500 dilution) for 20 min in the dark. Coverslips were again washed three times with PBS, then gently dipped four times into molecular biology-grade water before inversion onto one drop of Fluoro-Gel (with *N*-tris(hydroxymethyl)methyl-2-aminoethanesulfonic acid buffer, Electron Microscopy Sciences, #17985-30), and then allowed to air dry in the dark for at least 10 min. Slides were stored at 4°C until image collection on the LCCC Microscopy and Imaging Shared Resource (MISR) Leica SP8 microscope with the 63× oil objective at 1.52x magnification.

### DNase I and RNase H treatment

Cells were seeded at a density of 25,000 to 30,000 cells onto 18-mm-diameter #1.5 round coverslips (VWR, #101413-518) in 12-well dishes (day 1). They were allowed to attach to the coverslips for a full day (day 2). On the following day (day 3), the media were removed, and cells were washed three times with PBS and then fixed and permeabilized in 3.2% PFA with 0.2% Triton X-100 in PBS for 3 min at room temperature. Ten microliters of 1000 U of DNase I stock was added to 500 μl of DNase I buffer on cells for 10 min, or 2 μl of 5000 U of RNase H stock [New England Biolabs (NEB), M0297S] was added to 500 μl of RNase H buffer on cells for 5 min at 45°C. Cells were washed three times with PBS, and 3.2% PFA with 0.2% Triton X-100 was added again for 5 min after DNase I treatment or RNase H treatment. IF staining was completed as stated above.

### Evaluation of acrocentric chromosome copy number changes induced by TMZ resistance

As previously described, metaphase spreads from 42WT and 42R cells were prepared using a standard protocol (*17*). Chromosomes were stained with DAPI to determine total chromosome copy number and acrocentric chromosome copy number in each metaphase. Ratios of total chromosome number to acrocentric chromosome number in each metaphase were calculated and graphed in R.

### NanoBRET measurements for GW807982X (CLK2i)

CLK1, 2, and 4 NanoBRET assays were performed as previously described (*60*, *61*). Briefly, the N-terminal Nano Luciferase/CLK1 (NL-CLK1) fusion or C-terminal Nano Luciferase/CLK2 (CLK2-NL) fusion or C-terminal Nano Luciferase/CLK4 (CLK4-NL) fusion was encoded in pFN31K expression vector, including flexible Gly-Ser-Ser-Gly linkers between NL and CLK1, 2, or 4 (Promega Madison, WI, USA) (*62*). For cellular NanoBRET Target Engagement experiments, the NL-CLK1 or CLK2-NL or CLK4-NL fusion construct was diluted with carrier DNA-pGEM-3Zf (−) (Promega, Madison, WI, USA) at a mass ratio of 1:10 (mass/mass), before adding FuGENE HD (Promega, Madison, WI, USA). DNA:FuGENE complexes were formed at a ratio of 1:3 (μg DNA/μl FuGENE HD) according to the manufacturer’s protocol (Promega, Madison, WI, USA). The resulting transfection complex (1 part, volume) was then gently mixed with 20 parts (v/v) of human embryonic kidney (HEK) 293 cells (ATCC) suspended at a density of 2 × 10^5^ cells/ml in DMEM (Gibco) + 10% FBS (Seradigm/VWR). One hundred microliters of this solution was added to each well of a 96-well plate (Corning 3917) followed by incubation (37°C/5% CO_2_) for 24 hours. After 24 hours, the media were removed from the HEK293 CLK (NL-CLK1, CLK2-NL, or CLK4-NL) transfected cells and replaced with 85 μl of Opti-MEM media (Gibco). NanoBRET Tracer 5 (Promega, Madison, WI, USA) was used at a final concentration of 1.0 μM as previously evaluated in a titration experiment. A total of 5 μl per well [20× working stock of nanoBRET Tracer 5 (20 μM)] was added to all wells, except the “no tracer” control wells to which 5 μl per well of tracer dilution buffer alone was added. All inhibitors were prepared initially as concentrated stock solutions in 100% DMSO (Sigma-Aldrich). A total of 10 μl per well of the 10× chemical inhibitor stock solutions (final assay concentration, 1% DMSO) was added. For “no compound” and no tracer control wells, a total of 10 μl per well of Opti-MEM plus DMSO was added (final concentration, 1% DMSO). Ninety-six–well plates containing cells with NanoBRET Tracer 5 and inhibitors (100-μl total volume per well) were equilibrated (37°C/5% CO_2_) for 2 hours. To measure the NanoBRET signal, NanoBRET NanoGlo substrate at a ratio of 1:166 to Opti-MEM media in combination with extracellular NanoLuc Inhibitor diluted 1:500 [10 μl (30 mM stock) per 5 ml Opti-MEM plus substrate] was combined to create a 3× stock. A total of 50 μl of the 3× substrate/extracellular NanoLuc inhibitor was added to each well. The plates were read within 15 min (GloMax Discover luminometer, Promega, Madison, WI, USA) equipped with a 450 nM band-pass filter (donor) and a 600 nM low-pass filter (acceptor), using a 0.3-s integration time instrument using the “nanoBRET 618” protocol. Eleven concentrations of GW807982X were evaluated in competition with NanoBRET Tracer 5 in HEK293 cells transiently expressing CLK1, 2, and 4. Before curve fitting, the values are converted to mBRET units (×1000). Additional normalization of the NanoBRET assay data was performed by converting experimental values for respective concentrations of experimental inhibitors to relative percent control values [no compound (Opti-MEM + DMSO + Tracer 5 only) wells = 100% control, no tracer (Opti-MEM + DMSO) wells = 0% control]. The data were normalized to 0 and 100% inhibition control values and fitted to a four-parameter dose-response binding curve in GraphPad Software (version 7, La Jolla, CA, USA).

### Cell cycle analysis

For TMPyP4 treatment, on day 1, cells were seeded at 100,000 cells per well in six-well plastic tissue culture dishes 1 day before treatment with the indicated concentrations of drug. Fifty micromolar TMPyP4 was added on day 2, for an additional 48 hours. After 48 hours, cells were collected, washed with PBS, ethanol-fixed, stained with propidium iodide, and analyzed for cell sub-G1 (fragmented/apoptotic) DNA content and cell cycle profile. A total of 20,000 cells were acquired by flow cytometry on a Becton Dickinson Fortessa in the Flow Cytometry and Cell Sorting Shared Resource (FCSR). Files were modeled using ModFit software (Verity Software, Topsham, ME) to determine sub-G1, G_1_, S, and G_2_/M cell cycle stage. For CLK2i treatment, on day 1, cells were seeded at 200,000 cells per well in six-well plastic tissue culture dishes 1 day before treatment with the indicated concentrations of drug. Five micromolar CLK2i was added on day 2, for an additional 24 hours. After 24 hours, cells were collected, washed with PBS, ethanol-fixed, stained with propidium iodide, and analyzed for cell sub-G1 (fragmented/apoptotic) DNA content, cell cycle profile, and growth rate. A total of 20,000 cells were acquired by flow cytometry on a Becton Dickinson Fortessa. Files were modeled using ModFit software (Verity Software, Topsham, ME) to determine sub-G1, G_1_, S, and G_2_/M cell cycle stage.

### Nucleolar size and circularity detection and graphing

Images taken from the Leica SP8 microscope were opened in Fiji Is Just ImageJ (FIJI) (*63*), split into the channel to be analyzed, and converted from RGB to 8-bit images for analysis. In FIJI, the intensity threshold was set via Image -> Adjust -> Threshold (over/under 95). Next, the image was made into a binary form (Process -> Binary -> Make binary). Last, particles that met the threshold were quantified for area and circularity via Analyze -> Analyze particles. These readouts were then imported into R and graphed/analyzed using the R package Raincloud.

### Western blot analysis

Cells were lysed in radioimmunoprecipitation assay buffer supplemented with protease and phosphatase inhibitors (Roche, #4906837001) for protein extractions and separated by polyacrylamide gel electrophoresis using 4 to 12% gradient gels (Novex by Life Tech, #NP0321BOX) as described previously (*19*). They were then transferred onto Nitrocellulose membranes (Invitrogen, #IB23001) with the iBlot2 (Invitrogen, #IB21001) and probed with the following antibodies: EWSR1 (1:1000; Abcam, ab133288), CLK2 (1:1000; Sigma-Aldrich, HPA055366-100UL), and phosphorylated SR proteins (clone 1H4, 1:500; Millipore, #MABE50). β-Tubulin (1:5000; Sigma-Aldrich, #T7816) and β-actin (1:5000; Sigma-Aldrich, #A5316) were used as loading controls. Proteins were detected with horseradish peroxidase (HRP)–conjugated secondary antibodies [1:5000; GE Healthcare Life Sciences, #NA931-1ML (mouse) or #NA934-1ML (rabbit)] and enhanced chemiluminescent detection HyGLO Quick Spray Chemiluminescent (Denville Scientific, #E2400) using film (Denville Scientific, #E3212).

### Nuclear and cytoplasmic fractionation

On day 0, cells were seeded at 750,000 in a 10-cm dish. The following day, the media were aspirated and the plate was washed three times with cold PBS. Five hundred microliters of buffer A (250 mM sucrose, 20 mM Hepes, 10 mM NaCl, 1 mM EDTA, 1 mM EGTA, and 1.5 mM MgCl_2_ with 10 μl each of inhibitor: 1 M NaF, 200 mM sodium orthovanadate, 0.1 M phenylmethylsulfonyl fluoride, and 0.1 M dithiothreitol per 1 ml of buffer A made) was added to each dish, and the cells were scraped into a labeled Eppendorf tube. Lysate was then passed through a 26 5/8-gauge needle 10× and left on ice for 20 min. An aliquot was then taken out for whole-cell lysate (WCL), and the rest was centrifuged at 720*g* for 5 min at 4°C. The supernatant was transferred to a new tube and centrifuged at 720*g* for 3 min at 4°C to clean up the remaining nuclei. While the supernatant is spinning, the nuclear pellet was washed with 500 μl of buffer A plus inhibitors and pass through a new 26 5/8-gauge needle 10× more. The nuclear fraction was centrifuged at 3000 rpm for 10 min. As the nuclear fraction is spinning, the supernatant was collected from the cytoplasmic fraction and moved to a final tube. After the centrifugation of the nuclear fraction was finished, it was resuspended in 500 μl of 0.1% SDS in tris-buffered saline and sonicated. Western blots were then run on the WCL, nuclear (N), and cytoplasmic (C) fractions.

### High-resolution STED microscopy

STED images were acquired using a Leica SP8 3X STED microscope, a white-light laser for fluorescence excitation (470 to 670 nm), time-gated hybrid photomultiplier tubes (PMTs), and a Leica 100× (1.4 numerical aperture) STED White objective (Leica Microsystems Inc.). AF594 was excited with 575-nm excitation, with a back projected pinhole at 200 nm, and the fluorescence emission was collected at 610 nm with a line average of 4, frame accumulation of 2, and line accumulation of 1. Time gating of the emission signal from the PMT was set to a range of 0.7 to 6.5 ns for experiments involving the 775-nm depletion laser. *Z*-stacks were taken *X* = 24.890 nm, *Y* = 24.890 nm, and *Z* = 160.217 nm. The pinhole was set to a value of 0.7 airy units for all images. Image deconvolution was performed using Hyugens software (Scientific Volume Imaging B.V., The Netherlands) assuming an idealized STED point spread function.

### siRNA knockdown of EWSR1

Dharmacon individual set of four 2-nmol EWSR1 small interfering RNAs (siRNAs) were (LQ-005119-02-0002) resuspended in RNA reconstitution buffer provided. On day 0, 250 μl of Opti-MEM was warmed and added to 5 μl of siRNA (stock = 20 μM) and 7.5 μl of Trans-IT X2. The mixture was left to form micelles for 25 min. This was then added dropwise to six wells with 1% FBS media and 100,000 freshly plated cells (day 1). The cells were then adhered overnight, and media were changed the following morning. Cells were collected 48 hours later for either cell cycle analysis or Western blot, which allowed for 72 hours of transfection total.

### IHC staining on human GBM patient samples

IHC was performed for EWSR1 and CLK2 in deidentified human GBM tumor samples (table S2). Five-micrometer sections from formalin fixed paraffin embedded tissues were deparaffinized with xylenes and rehydrated through a graded alcohol series. Heat-induced epitope retrieval was performed by immersing the tissue sections at 98°C for 20 min in 10 mM citrate buffer (pH 6.0) with 0.05% Tween 20. IHC was performed using a HRP-labeled polymer from Agilent Technologies (K4003) according to the manufacturer’s instructions. Briefly, slides were treated with 3% hydrogen peroxide and 10% normal goat serum for 10 min each and exposed to primary antibodies for EWSR1 (1:400; Abcam, ab133288) or CLK2 (1:800; Human Protein Atlas, HPA055366) overnight at 4°C. Slides were exposed to the appropriate HRP-labeled polymer for 30 min and 3,3′-diaminobenzidine chromagen (Dako) for 5 min. Slides were counterstained with hematoxylin (Fisher, Harris Modified Hematoxylin), blued in 1% ammonium hydroxide, dehydrated, and mounted with Acrymount. Consecutive sections with the primary antibody omitted were used as negative controls.

### RNA isolation

A total of 750,000 of various cells were seeded on day 1. The following day, cells were washed three times with PBS, trypsinized, and pelleted into an Eppendorf tube. Then, 4 ml of TRIzol was added to each tube and incubated at room temperature for 5 min. One milliliter of each sample was aliquoted into four separate tubes. Two hundred microliters of chloroform was added to each tube and vortexed briefly to mix. Samples incubated at room temperature for 5 min were briefly vortexed and then centrifuged at 12,000*g* for 10 min at 4°C. Five hundred microliters of the clear upper layer was collected from each tube and pooled into two 1-ml Eppendorf tubes where 500 μl of isopropanol was added and mixed thoroughly by multiple inversions. Samples were incubated for 15 min at room temperature and then centrifuged for 15 min at 12,000*g* at 4°C. The supernatant was then discarded, and the pellet was washed with 750 μl of 80% ethanol by multiple inversions. The sample was centrifuged for 5 min at 4°C at 12,000*g*. The pellet was again washed with 80% ethanol by multiple inversions and centrifuged the same way as before. The supernatant was removed and discarded, and a second quick spin was done to remove any residual contaminating liquid. The RNA pellet was then air-dried for 10 min before being resuspended in 100 μl of nuclease-free water. Total RNA was quantified using a NanoDrop 2000, and quality assurance was performed by the Agilent BioAnalyzer.

### Illumina short-read sequencing

Twenty-microgram aliquots of total RNA were used for preparation of indexed, 150-base paired-end sequencing libraries using the TruSeq Stranded Total RNA Library Prep Kit (Illumina). Sequencing was performed on the Illumina NextSeq 550 instrument in the Genomics and Epigenomics Shared Resource (GESR) at a minimum of 65 M reads per sample.

### Nanopore full-length cDNA sequencing

Twenty-microgram aliquots of total RNA were diluted in 100 μl of nuclease-free water, and poly-A was selected using NEXTflex Poly(A) Beads (BIOO Scientific, catalog no. NOVA-512980). Resulting poly-A RNA was quantified, and 50-ng aliquots were transferred to thin-walled Eppendorf polymerase chain reaction (PCR) tubes. The biological poly-A RNA and a synthetic control (Lexogen SIRV Set 3, 0.5 ng) were prepared for cDNA synthesis and nanopore sequencing following the ONT SQK-PCS108 kit protocol with a few exceptions. During the reverse transcription step, Superscript IV was used, and the reverse transcription incubation time was increased to 15 min. After reverse transcription, PCR was performed using LongAmp Taq Master Mix (NEB) under the following conditions: 95°C for 30 s, 10 cycles (95°C for 15 s, 62°C for 15 s, and 65°C for 15 min) and 65°C for 15 min, hold at 4°C. The resulting cDNA libraries were quantified, and sequencing libraries were prepared using 700 ng of cDNA following the standard protocol for SQK-PCS108 (one-dimensional sequencing). Sequencing was performed on the GridION platform using ONT R9.4 flow cells and the standard MinKNOW protocol script (NC_48hr_sequencing_FLO-MIN106_SCK-PCS108).

### Nanopore full-length sequencing analysis with short-read Illumina correction

Albacore workflow (version 2.1.3) was used for base-calling cDNA data. Using minimap2 (*27*) with recommended parameters (-ax splice -uf -k14), reads were aligned to the GRCh38 (*64*) human genome reference to investigate possible novel isoforms. The latest GRCh38 assembly was used as the reference genome in all alignments. Long reads were mapped to the reference using minimap2 in spliced alignment mode with recommended parameters (-ax splice --junc-bed). Similarly, short reads were aligned to the reference with spliced aligner HISAT2 (*65*). To use both the long- and short-read alignments together in assembly, StringTie (*66*) was used in “--mix” mode to identify and quantify isoforms, some of which are novel (*67*). These novel and annotated isoforms were then analyzed for potential isoform switching with IsoformSwitchAnalyzeR (*29*).

The genes that were identified to have statistically significant changes in isoform expression levels between cell lines were filtered through three stages:

1) The dominant open reading frame (ORF) of the isoforms was identified by IsoformSwitchAnalyzeR, and switches between isoforms that result in the same ORF sequence were filtered out.

2) To analyze the switching events that possibly correlate with TMZ resistance, only the genes that show switching in both 42R and T98G cell lines when compared to the 42WT cell line were included.

3) With only two replicates per cell line, the gene switch *Q* values generated by IsoformSwitchAnalyzeR cannot confidently assess biological variation. To eliminate any potential false positives due to low number of replicates, a secondary differential splicing analysis was conducted with SUPPA2 (a tool that exploits between-replicate variability and can accurately evaluate percent spliced in (PSI) values using two replicates per condition) (*30*). Stage (2) was also applied to SUPPA2 results, and only the overlapping switch genes between IsoformSwitchAnalyzeR and SUPPA2 results were included.

The final list of genes after filtering was investigated for mutation enrichment [using the genome arithmetic framework of BEDTools (*68*)] and the downstream pathways that were potentially affected. Files and codes associated with this analysis can be found at https://github.com/timplab/Drug_resistant_GBM.

### DNA isolation for WGS

A total of 750,000 cells were collected for each cell line, where DNA was isolated using the DNeasy Blood and Tissue Kit (Qiagen, catalog no. 69504). Briefly, cells were centrifuged and resuspended with proteinase K and buffer AL for 10 min at 56°C. Two hundred microliters of ethanol was then added and mixed thoroughly by vertexing. DNA was added to the DNeasy Mini spin column, centrifuged, and flow-through discarded. DNA was washed in the DNeasy column with buffer AW1 once and buffer AW2 once as well. Membrane was then dried by centrifugation with all flow-through being discarded. DNA was eluted using 200 μl of buffer AE after 1-min incubation at room temperature.

### WGS using Illumina TruSeq DNA PCR-free library prep kit

The Illumina TruSeq DNA PCR-Free Library Prep Kit was used per manufacturer’s instructions by the GESR. Paired-end, indexed libraries for human WGS were constructed from 1.0 μg of gDNA using the TruSeq DNA PCR-Free Library Prep Kit (Illumina, San Diego) according to the manufacturer’s instructions. Briefly, DNA was fragmented using a Covaris M220 Focused-ultrasonicator (Covaris, Woburn, MA) using settings for a 350-bp insert size. Library quality was assessed with a BioAnalyzer 2100 using the High Sensitivity DNA kit (Agilent Technologies, Santa Clara, CA). The libraries were quantified using the Kapa Library Quantification Kit Illumina Platforms (Kapa Biosystems, Boston, MA). The denatured and diluted libraries were sequenced on a NextSeq 550 System (Illumina) using v2.5 High Output 300 cycle kit with 1% PhiX to an average sequencing depth of 50× coverage.

### WGS analysis

Raw data were first demultiplexed into three groups, which were 42WT, 42R, and T98G, and each group had two biological duplicates. All raw sequence data (fastq files) were run through fastQC v0.11.7 for quality check, and lower-quality reads (*Q* < 20) were eliminated. Cutadapt v2.1 was used for adapter trimming, and read length less than 20 bp after trimming was filtered. Processed reads were then aligned to GRCh38 reference sequence using the bwa v0.7.17 paired-end mode. Mutation detection was conducted in Genome Analysis Toolkit (GATK) v4.1.0.0 (*69*) following the best practice for variant calling workflow. The consensus assembly for each sample was created by bcftools, and then the Quadron methodology (*19*) was used to detect stable G4 structures. Unique G-Quad sequence with *Q* > 19 was extracted from each group for further analysis. COSMIC Mutational Signatures version 3.2 was used to classify single base substitution (SBS), doublet base substitution (DBS), and small insertions and deletions (ID) in cell line and GLASS consortium WGS data using SigProfiler (*70*). Tumor mutation burden was calculated using the formula (# mutation in ID, SBS, DBS/# bases covered by WGS × 1,000,000).

## Notes

View/request a protocol for this paper from *Bio-protocol*.
