# Supplementary material for: Temozolomide-induced guanine mutations create exploitable vulnerabilities of guanine-rich DNA and RNA regions in drug-resistant gliomas
Source: Sci Adv. 2022 Jun 22;8(25):eabn3471. doi: 10.1126/sciadv.abn3471 (PMC9216507; doi:10.1126/sciadv.abn3471)
Supplement: Supplementary file 1 — Figs. S1 to S9 Tables S1 and S2 [file sciadv.abn3471_sm.pdf]

Science Advances
AAAS

Supplementary Materials for

Temozolomide-induced guanine mutations create exploitable vulnerabilities of guanine-rich DNA and RNA regions in drug-resistant gliomas

Deanna M. Tiek *et al.*

Corresponding author: Deanna M. Tiek, deanna.tiek@northwestern.edu; Rebecca B. Riggins, rbr7@georgetown.edu

Sci. Adv. **8**, eabn3471 (2022)
DOI: 10.1126/sciadv.abn3471

The PDF file includes:

Figs. S1 to S9
Tables S1 and S2
Legend for table S3

Other Supplementary Material for this manuscript includes the following:

Table S3

Table of contents page

Supplemental Fig S1	3
Supplemental Fig S2	5
Supplemental Fig S3	7
Supplemental Fig S4	9
Supplemental Fig S5	11
Supplemental Fig S6	13
Supplemental Fig S7	15
Supplemental Fig S8	16
Supplemental Fig S9	18
Supplementary Table 1	20
Supplementary Table 2	21
Supplementary Table 3	22, excel sheet uploaded separately

Supplemental Figure 1

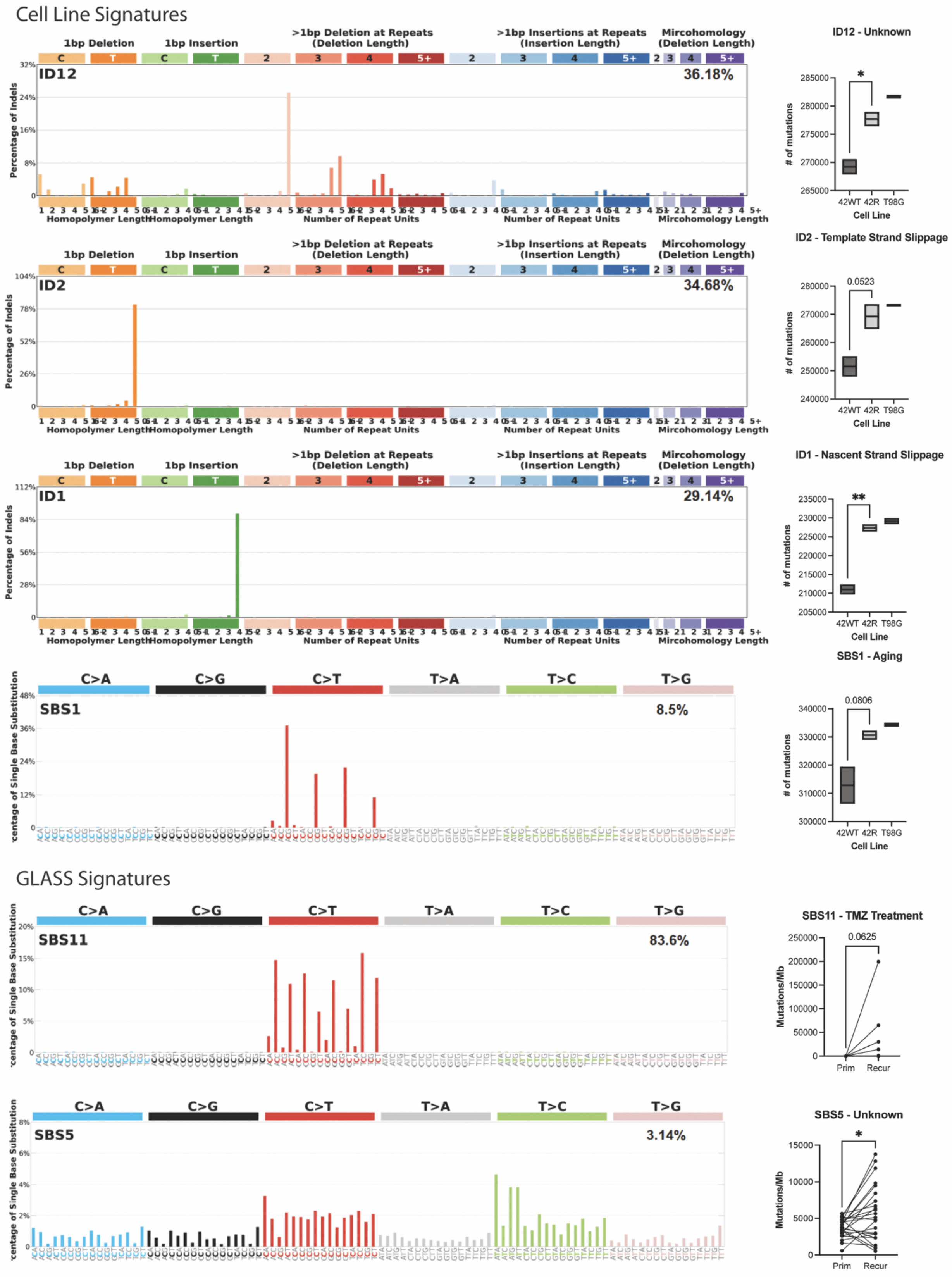

Supplementary Figure 1. Expanded mutational signature characterization. Cell line (42WT, 42R, T98G) and GLASS matched patient samples from primary (Prim) and recurrent (Recur) tumors were analyzed to determine significant single base substitution (SBS) mutational signature profiles. * $p<0.05$, ** $p<0.001$

Supplemental Figure 2

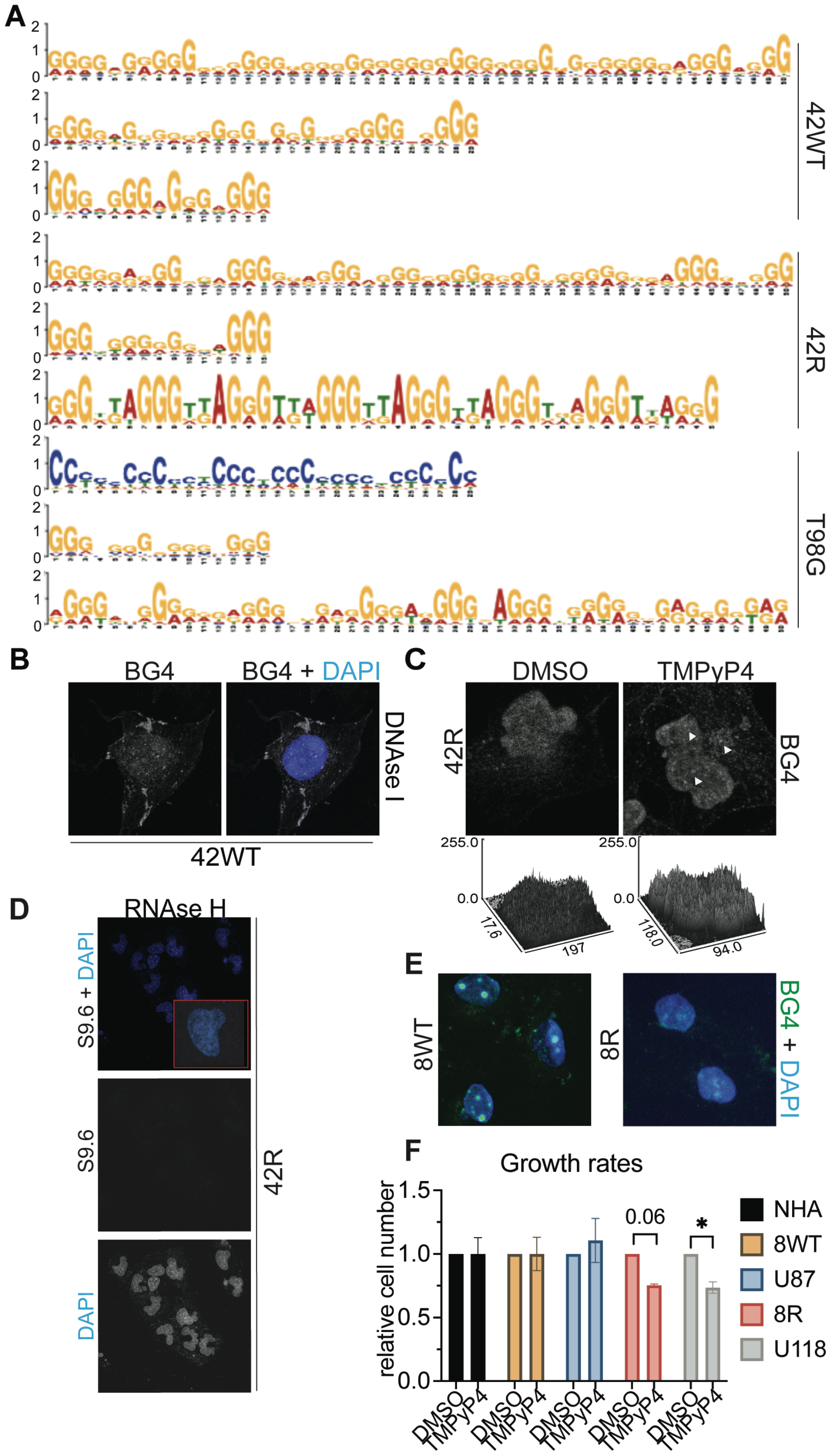

Supplementary Figure 2. Specificity controls for BG4 and TMPyP4. (**A**) MEME-predicted enriched motifs from Quadron-derived unique acrocentric chromosome sequences between 42WT (E-value top; 1.4e-231, bottom; 9.4e-013), 42R (E-value 7.9e-206), and T98G (E-value top; 9.3e-198, bottom; 7.2e-013) cell lines. (**B**) DNase I treatment of 42WT cells abrogates BG4 G-quadruplex antibody staining. (**C**) Treatment with 50 μM TMPyP4 vs. DMSO for 24 hour in 42R increases discrete punctate staining of BG4 and increased surface plot height. Nuclear surface plot of signal intensity depicted on the right was generated by FIJI. (**D**) RNase H treatment of 42R cells abrogates S9.6 R-loop antibody staining. (**E**) IF of G4s via BG4 antibody in isogenic TMZ-S/R (8WT/R) cell lines. (**F**) Crystal violet staining of normal human astrocyte (NHA), TMZ-sensitive (8WT, U87) and TMZ-resistant (8R, U118) treated with 50 μM TMPyP4 for 48 hours. * $p<0.05$

Supplemental Figure 3

A

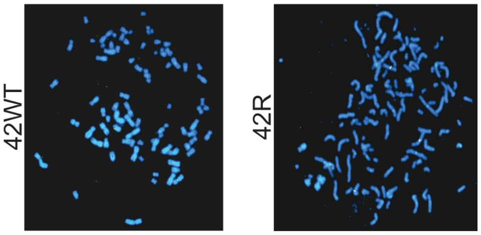

B

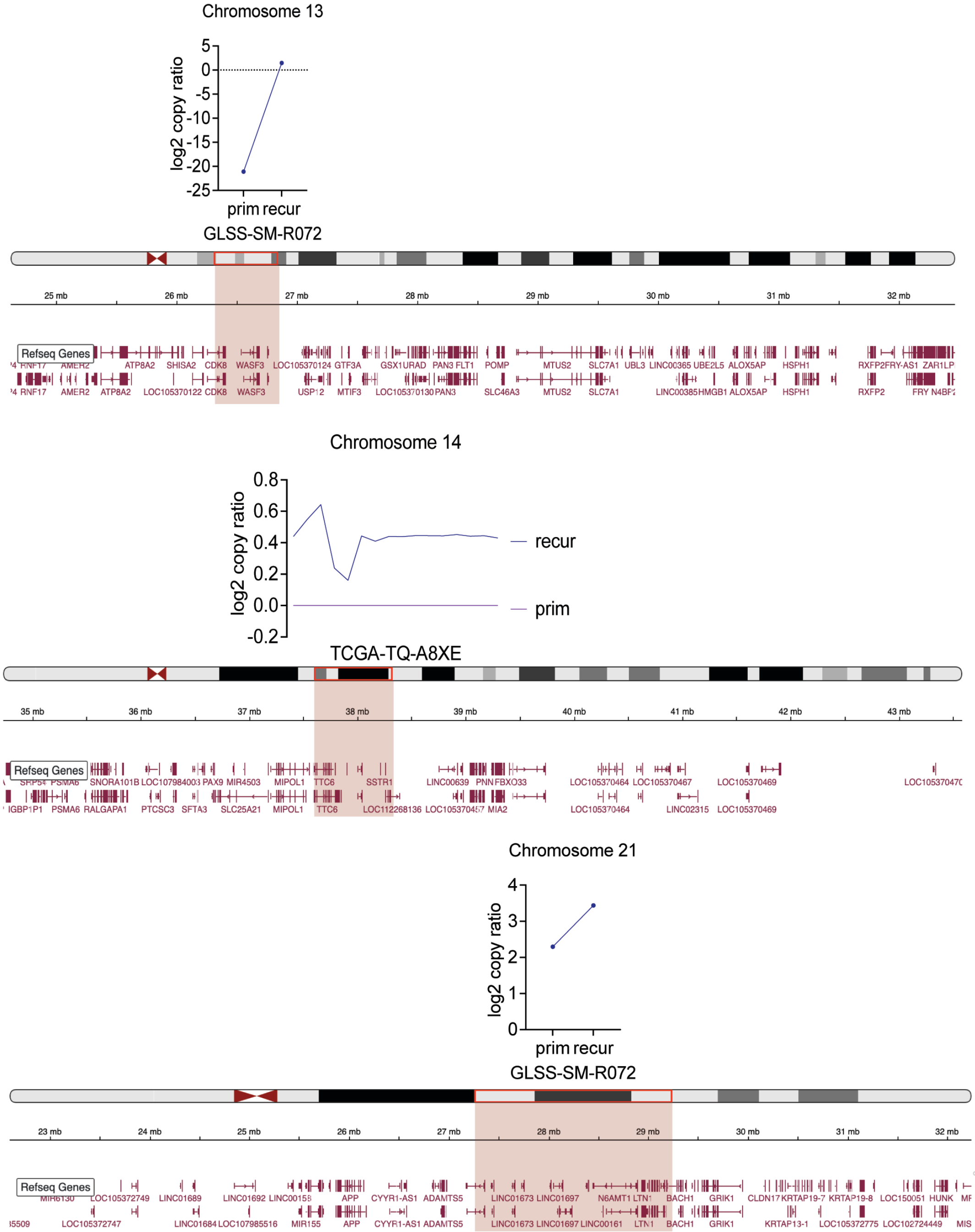

Supplemental Figure 3. Representative acrocentric chromosome changes. (**A**) Representative images of metaphase spreads in denoted cell lines. (**B**) Examples of increases (change in log2 copy ratio >/= 2) in acrocentric chromosomes 13, 14, and 21 in GLASS patient samples of matched primary (prim) and recurrent (recur) tumors.

Supplemental Figure 4

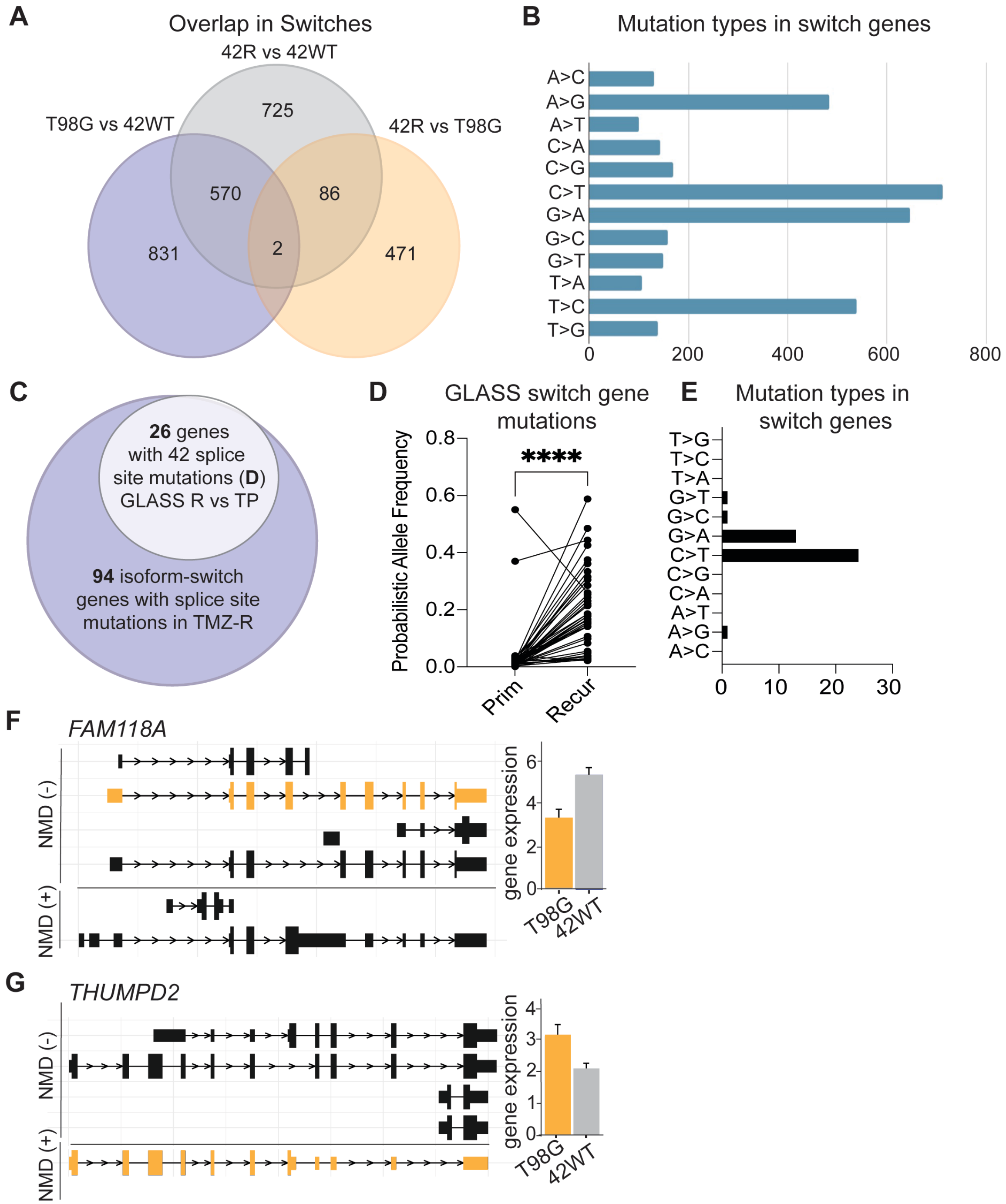

Supplementary Figure 4. Additional nanopore and WGS mutation results. (A) Venn Diagram of the overlap in switched isoforms between the indicated cell lines. (**B**) Mutation type abundance in switched genes that also have splicing mutations. (**C**) Overlap of 26 genes from the GLASS

matched primary (TP) and recurrent (R) patient samples with the 94 isoform-switch genes from cell line data. (**D**) Probabilistic allele frequency of the 42 splice site mutations from the 26 overlapping genes in 3C. (**E**) Mutation type from 3D. (**F**) *FAM118A* and (**G**) *THUMPD2* gene isoform changes between NMD-sensitive and -insensitive in TMZ-sensitive (42WT) versus TMZ-resistant (T98G) cells.

Supplemental Figure 5

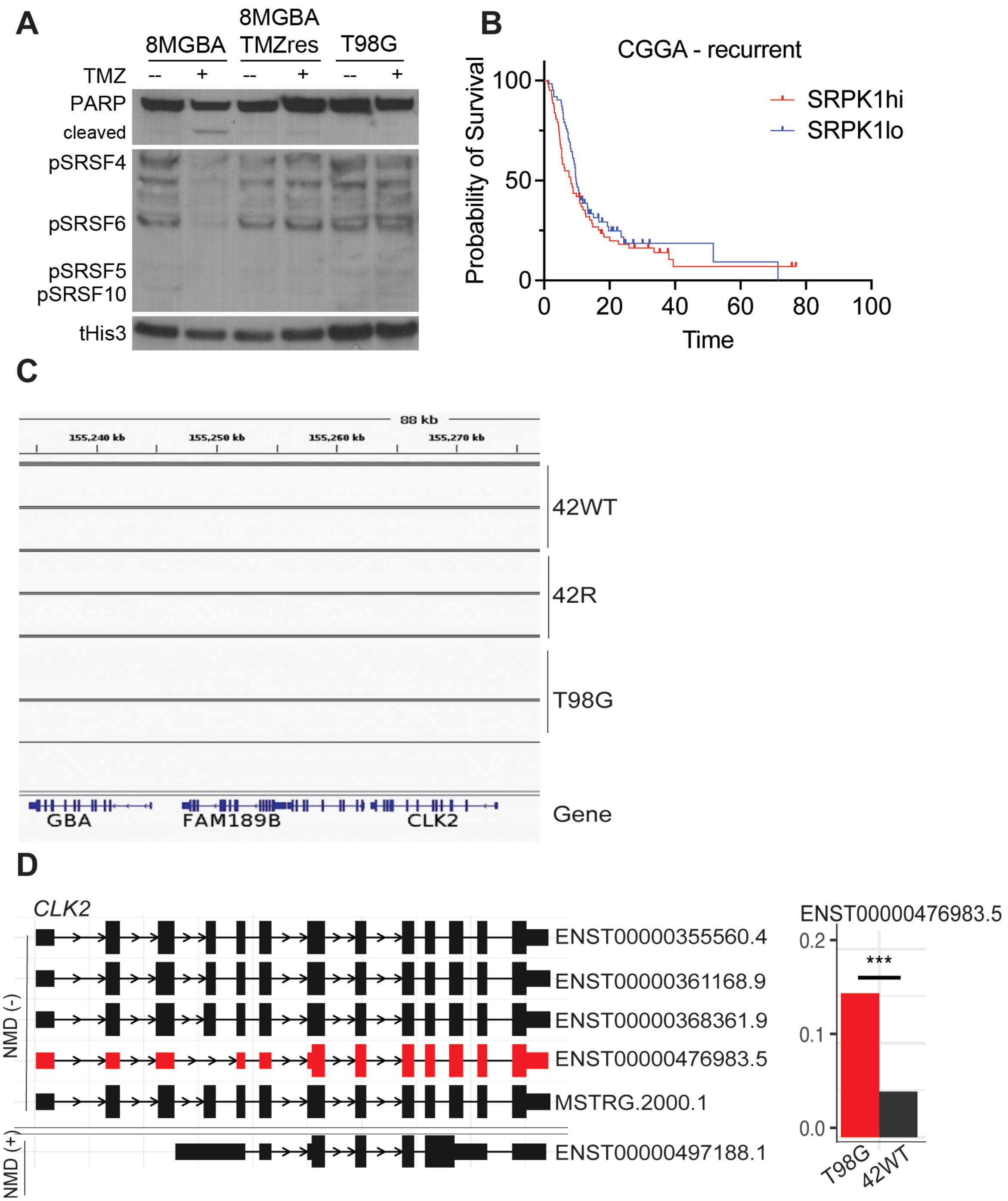

Supplementary Figure 5. Additional characterization of pSR changes and CLK2 characterization. (**A**) Western blot of additional isogenic TMZ-S/R lines (8WT/8R) and TMZ-R line (T98G) +/- 100 µM TMZ treatment for 72 hr. PARP/cleaved showing apoptosis, cl IH4

showing pSR changes, tHis3 loading control. (**B**) GlioVis analysis of SRPK1 KM plot in CGGA GBM recurrent patients. (**C**) WGS data showing no *CLK2* mutations in the indicated cell lines. (**D**) Nanopore data showing CLK2 isoform changes where the red isoform denotes the isoform with increased usage in the TMZ-R line T98G as compared to 42WT, quantified on the right. ***$p<0.0005$

Supplemental Figure 6

A

Kinase	Nanosyn % inhibition 100 nM	Nanosyn % inhibition 1.0 µM	Discover X PoC 1µM	Discover X K_D nM	RBC IC_{50}
CLK1			2.9	26	10 nM
**CLK2**	**81%**	**96%**	**8.3**	**4.9**	**0.73 nM**
CLK3	4%	22%	89	n/a	nt
CLK4			27	140	8.8 nM
DYRK1A	8%	22%	73	nt	nt
DYRK1B	5%	39%	81	nt	nt
DYRK2	1%	6%	100	nt	nt
DYRK3			n/a	nt	> 10 µM
DYRK4			n/a	nt	> 10 µM
HIPK1	20%	62%	20	3600	> 10 µM
HIKP2			27	530	nt
HIPK3			74	nt	nt
HIPK4	4%	24%	76	nt	nt
SRPK1	-1%	-1%	100	nt	nt
SRPK2			100	nt	nt
MSSK1	0%	0%	100	nt	nt
PRP4			100	nt	nt
STK16			6.9	210	100 nM
ERK8			4.4	35	120 nM
NEK6	0%	2%	11	84	> 10 µM
NEK7	3%	2%	19	140	> 10 µM

B

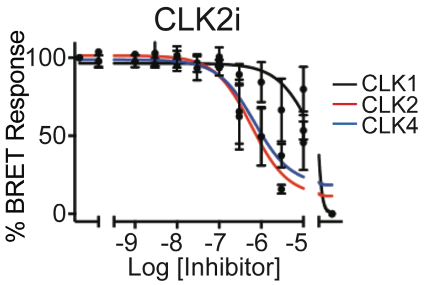

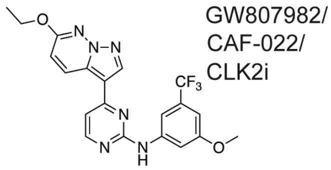

GW807982/
CAF-022/
CLK2i

C

NanoBRET IC50s
CLK1 IC50 => 10,000 nM
CLK2 IC50 = 580 nM
CLK4 IC50 = 690 nM

D

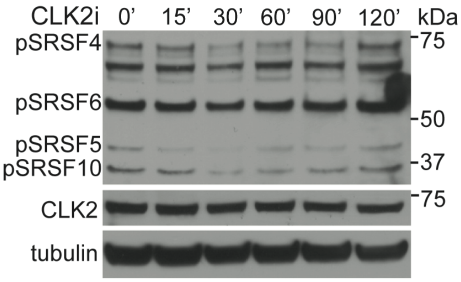

E

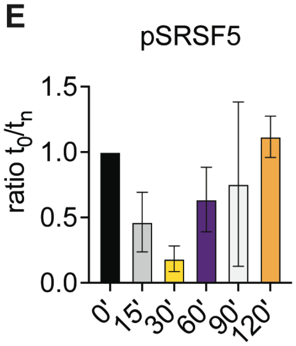

F

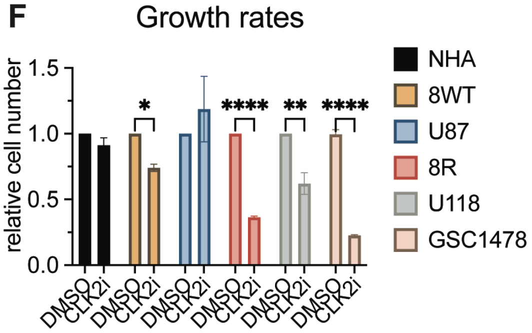

Supplementary Figure 6. **Characterization of a novel CLK2 inhibitor (GW807982X, CLK2i).** (**A**) Commercial kinase binding and activity assays were performed at NanoSYN (data included are percent inhibition at 100 nM and 1 µM CLK2i; assays were conducted at [ATP] = K_m^{app} of each kinase), DiscoverX (data included are K_D values), and Reaction Biology Corporation (data expressed are IC_{50} values of kinase inhibition; assays were conducted at 10 µM ATP). "nt" denotes that the compound was not tested in these assays. (**B**) NanoBRET assay (77) of CLK2i (CAF-022) with multiple CLK family members in live cells, structure of CLK2i/CAF-022/GW907982X. (**C**) NanoBRET IC_{50} values determined for CLK1, 2, and 4. (**D**) Western blot of pSR changes over a time-course of CLK2i treatment in 42R cells (**E**) quantification of maximal pSRSF5 hypophosphorylation from 3 independent replicates as performed in (**D**) where n=time on the x-axis. CLK2i, GW807982X and CAF-022 are all names for the same compound. The NanoSYN data shown in (A) were originally published in (39). (**F**) Crystal violet staining of normal human astrocyte (NHA), TMZ-sensitive (8WT, U87) and TMZ-resistant (8R, U118, GSC1478) treated with 5 µM CLK2i for 48 hours. * $p<0.05$, ** $p<0.001$, **** $p<0.0001$.

Supplemental Figure 7

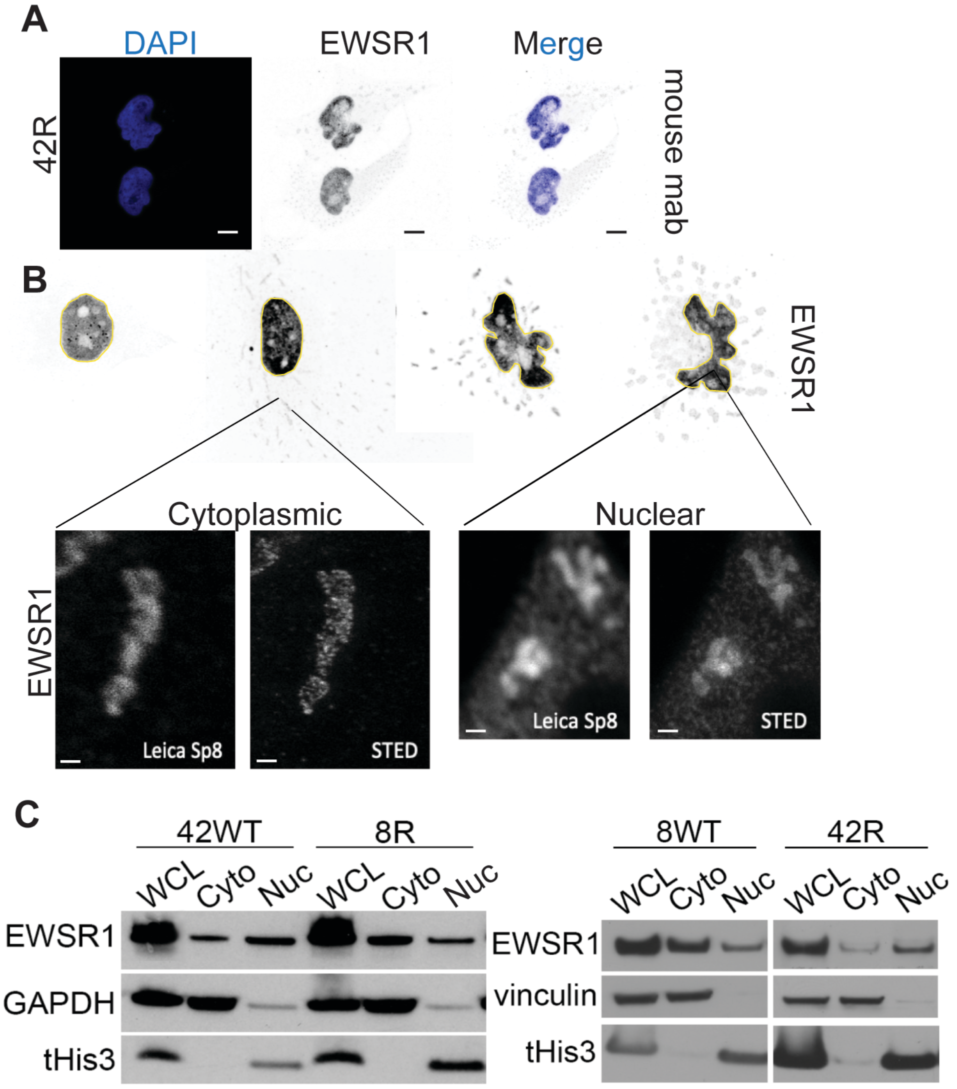

Supplementary Figure 7. Controls for EWSR1 IF, aggregation, and STED imaging. (**A**) 42R cells stained with the EWSR1 mouse monoclonal antibody (mAb, Santa Cruz sc48404) and DAPI, scale bar 10 µm. (**B**) Staining with the EWSR1 rabbit mAb in 42WT, 42WT-treated with 100 µM

TMZ for 72 hr, 42R, and T98G cells, higher magnification of nuclear EWSR1 staining is shown in both Leica Sp8 confocal and STED images, scale bar 2.5 µm. (**C**) Western blot of fractionated TMZ-S (42WT, 8WT) and TMZ-R (42R, 8R) cell lines. WCL; whole cell lysate, Cyto; cytoplasmic fraction; Nuc; nuclear fraction

Supplemental Figure 8

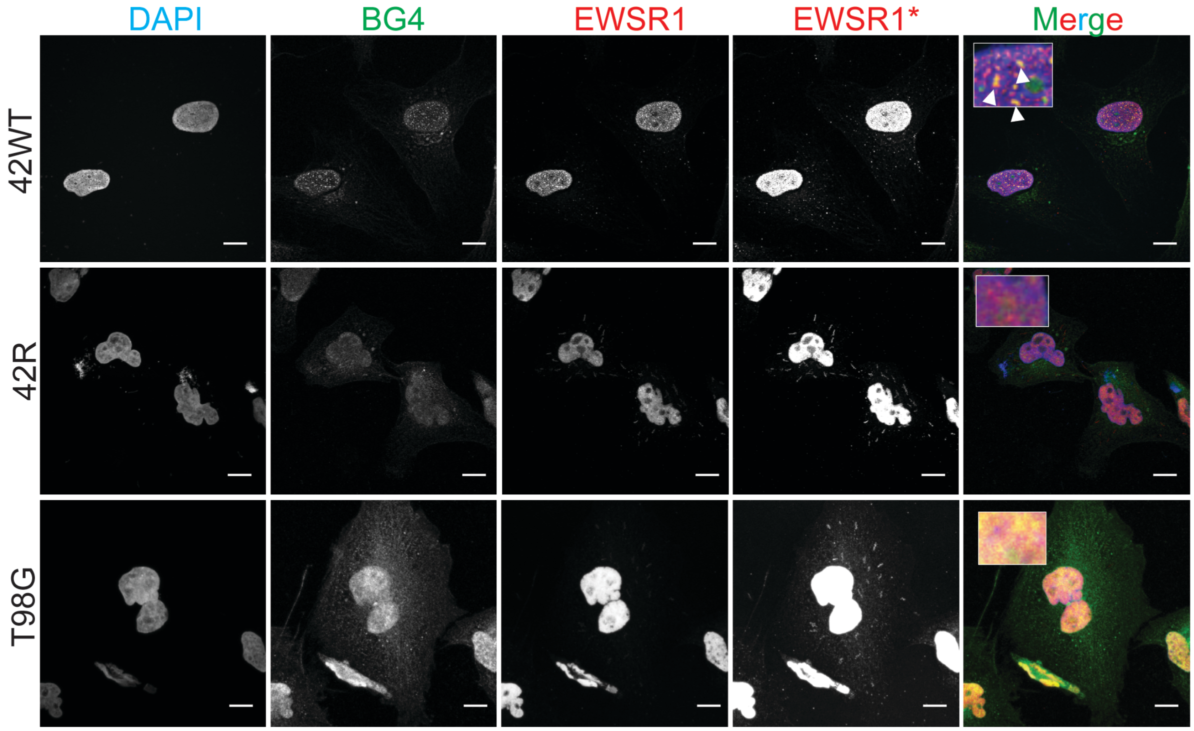

Supplementary Figure 8. Controls for EWSR1 colocalization with G4s. Co-stain of the G4 antibody BG4 with EWSR1 in 42WT, 42R, T98G cells. EWSR1* shows enhanced images to permit visualization of cytoplasmic aggregates. Merged panels show an inset with higher magnification of BG4 and EWSR1 localization, arrows in 42WT show yellow colocalization of EWSR1 and BG4 staining. Scale bar 50 µm.

Supplemental Figure 9

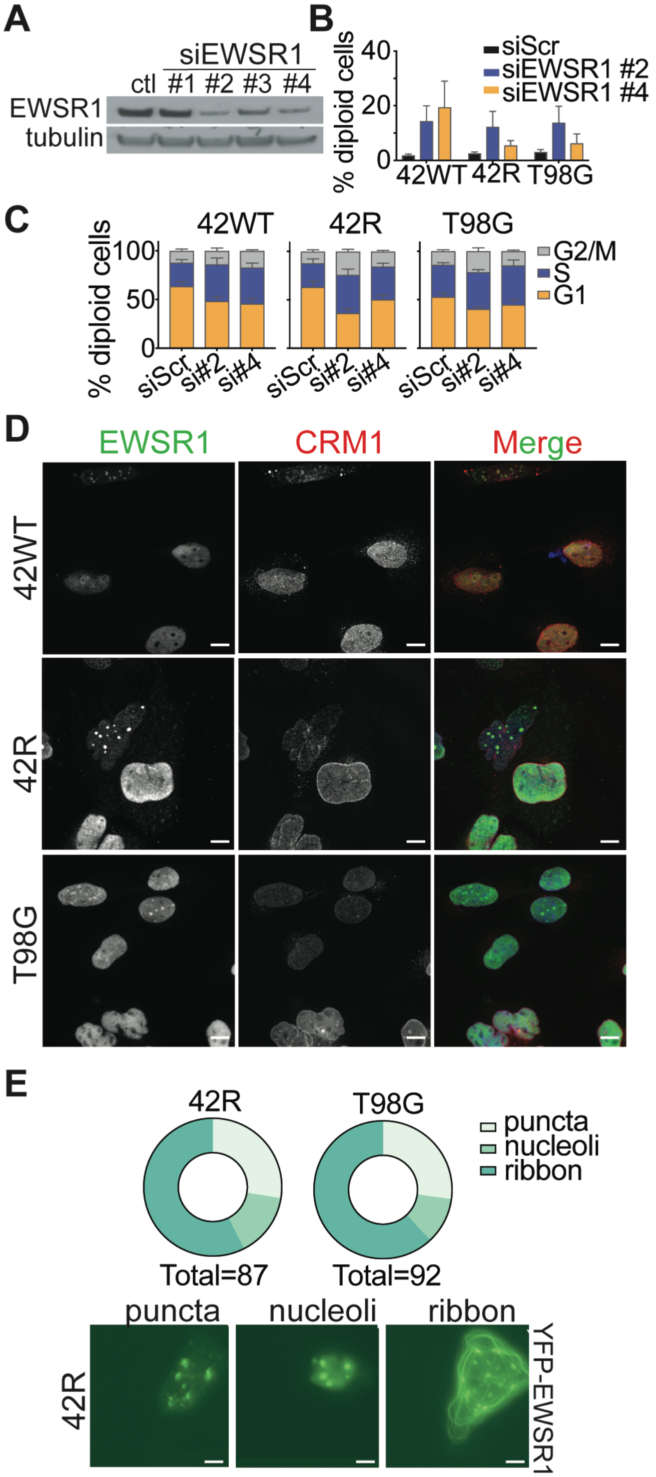

Supplementary Figure 9. Controls for endogenous EWSR1 depletion and nucleo-cytoplasmic trafficking, and ectopic YFP-EWSR1 cytoplasmic aggregation. (**A**) Western blot of EWSR1

knockdown with four different siRNAs. (**B**) FACS analysis of subG1 DNA content following 72 hours of EWSR1 depletion. (**C**) Cell cycle profile of cells analyzed in (**B**) for 42WT, 42R, T98G cells; three biological replicates. (**D**) Treatment with 25nM Leptomycin B (CRM1 inhibitor; LMB) in 42WT, 42R, and T98G cells for 24 hr. (**E**) Quantification of the phenotype resulting from overexpression of YFP-EWSR1 phenotype in 42R n=87 cells, and T98G n=92 cells. Representative images below for “puncta”, “nucleoli”, and “ribbon” phenotypes. Scale bars in (**D)** 50 µm and (**E**) 10µm.

Supplementary Table 1. List of significant genes with both splice site mutations and isoform usage changes

AC002074
AC010615
AC020765
AC138207
ACAT1
ADNP2
AK5
AKIP1
AKR1C1
ANAPC13
ANAPC5
AP2S1
APIP
ARFGEF2
BMP2K
BROX
CARM1
CCDC50
CDK5RAP2
CDKN2D
CENPU
CHID1
DDR2
DNAJC3
EPOR
ERRFI1
ESYT2
FAM117B
FAM118A
FECH
FMN1
GFER
GFOD1
HECTD2
HLCS
HNRNPU
ICMT
KANK1
L3MBTL2
LCLAT1
LDLRAD3
LINC01943
LRIG1
LTN1
MAP3K7
METRN
MINDY3
MMAB
MRPL11
MYO10
NBPF12
NCDN
NUDT19
NUPR1
PARD3
PGGT1B
PGM2
PRELID2
PREP
PXDC1
RANGRF
RAVER2
RECQL
RELCH
RGL3
RMI2
RSU1
RUNX1
SCAND1
SEPTIN7P14
SFXN4
SH3D19
SLC2A10
SLC9A3R2
SNX1
SSNA1
SULT1A1
SULT1A2
THUMPD2
TNFRSF11A
TOP1MT
TPM4
TRAPPC10
UBAC2
UBAP1
VANGL1
VIT
ZDHHC7
ZFP64
ZFR
ZNF595
ZNF652
ZNF738
ZNF764

Supplementary Table 2. Demographic, clinical, and pathological characteristics of GBM clinical samples

Cohort 1: GU

ID #	[a]Diagnosis	Age	Sex	Cytoplasmic EWSR1 aggregation
^Gu-1	GBM	89	Male	Yes
^Gu-2	GBM	69	Female	Yes
^Gu-3	GBM	35	Female	Yes
Gu-4	GBM	44	Male	No
Gu-5	GBM	68	Female	No
Gu-6	GBM	54	Male	No
*Gu-7	AA	54	Female	No
*Gu-8	GBM	54	Female	Yes
Gu-9	GBM	77	Male	No
Gu-10	AA	62	Male	No
Gu-11	AA	36	Female	No
Gu-12	MB	49	Female	No
^Gu-13	GBM	50	Male	Yes
Gu-14	GBM	59	Female	No
^Gu-15	GBM	81	Female	Yes
Gu-16	MB	25	Male	No
Gu-17	MB	50	Female	No
^Gu-18	GBM	64	Male	Yes
Gu-19	GBM	40	Male	No
Gu-20	GBM	76	Female	Yes
Gu-21	GBM	59	Female	Yes

^denotes representative image shown in Figure 6A, *denotes same patient and surgical event
[a]GBM, glioblastoma; AA, anaplastic astrocytoma; MB, medulloblastoma

Cohort 2: JTCC

ID #	[a]Diagnosis	Age	Sex	Race	[b]Ethnicity	[c]OS (mos)	[d]MGMT	[e]EGFR	[f]IDH1/2
S1	GBM	54	Male	White	N-H/L	19	Me	Amp, vIII+	WT
S2	GBM	49	Male	White	N-H/L	17	Nd	Amp, vIII+	WT
S3	GBM	56	Female	White	N-H/L	42	Me	No Amp	WT
S4	GBM	44	Male	White	N-H/L	12	Nd	Amp, vIII+	WT

[a]GBM, glioblastoma
[b]N-H/L, not Hispanic or Latino
[c]OS (mos), overall time of survival in months
[d]Me, MGMT methylation detected; Nd, not detected
[e]Amp, EGFR amplification detected; vIII+, EGFRvIII detected; no Amp, no amplification detected
[f]WT, negative for IDH1/2 mutation

Supplementary Table 3. ***ATRX*** **mutations in GBM cell lines detected by whole-genome sequencing.** Excel sheet uploaded separately shows mutations unique to each cell line and common across all cell lines.
